# An African loss-of-function *CACNA1C* variant p.T1787M associated with a risk of ventricular fibrillation

**DOI:** 10.1038/s41598-018-32867-4

**Published:** 2018-10-02

**Authors:** Malorie Blancard, Amal Debbiche, Koichi Kato, Christelle Cardin, Guichard Sabrina, Estelle Gandjbakhch, Vincent Probst, Michel Haissaguerre, Fabrice Extramiana, Mélèze Hocini, Geoffroy Olivier, Antoine Leenhardt, Pascale Guicheney, Jean-Sébastien Rougier

**Affiliations:** 1 INSERM, UMR_S1166, Paris, France; 2 Sorbonne University, UPMC Univ Paris 06, Institute of Cardiometabolism and Nutrition (ICAN), Paris, France; 3 University Hospital Rangueil, Toulouse, France; 4 University of Bern, Institute of Biochemistry and Molecular Medicine, Bern, Switzerland; 5 AP-HP, Hôpital Pitié-Salpêtrière, Département de Cardiologie, Paris, France; 6 CHU Nantes, L’institut du thorax, Service de Cardiologie, Nantes, France; 7 L’Institut de Rythmologie et Modélisation Cardiaque (LIRYC), Université de Bordeaux, Bordeaux, France; 8 Inserm U1045 CRCTB, Université de Bordeaux, Bordeaux, France; 9 AP-HP, Hôpital Bichat, Département de Cardiologie, Centre de Référence des Maladies Cardiaques Héréditaires, Paris, France; 10 CHU Sud Réunion, La Réunion, France

## Abstract

Calcium regulation plays a central role in cardiac function. Several variants in the calcium channel Ca_v_1.2 have been implicated in arrhythmic syndromes. We screened patients with Brugada syndrome, short QT syndrome, early repolarisation syndrome, and idiopathic ventricular fibrillation to determine the frequency and pathogenicity of Ca_v_1.2 variants. Ca_v_1.2 related genes, *CACNA1C*, *CACNB2* and *CACNA2D1*, were screened in 65 probands. Missense variants were introduced in the Ca_v_1.2 alpha subunit plasmid by mutagenesis to assess their pathogenicity using patch clamp approaches. Six missense variants were identified in *CACNA1C* in five individuals. Five of them, A1648T, A1689T, G1795R, R1973Q, C1992F, showed no major alterations of the channel function. The sixth C-terminal variant, Ca_v_α_1c_-T1787M, present mostly in the African population, was identified in two patients with resuscitated cardiac arrest. The first patient originated from Cameroon and the second was an inhabitant of La Reunion Island with idiopathic ventricular fibrillation originating from Purkinje tissues. Patch-clamp analysis revealed that Ca_v_α_1c_-T1787M reduces the calcium and barium currents by increasing the auto-inhibition mediated by the C-terminal part and increases the voltage-dependent inhibition. We identified a loss-of-function variant, Ca_v_α_1c_-T1787M, present in 0.8% of the African population, as a new risk factor for ventricular arrhythmia.

## Introduction

Cardiac channelopathies are genetic disorders associated with an increased risk of ventricular arrhythmia and sudden death (SD) in young individuals with a structurally normal heart. They include long QT syndrome (LQTS), Brugada syndrome (BrS) as well as other more rare diseases such as short QT syndrome (SQTS), early repolarisation syndrome (ERS), idiopathic ventricular fibrillation (IVF) and short-coupled torsades de pointes (scTdP)^1^. An important aspect of arrhythmia pathophysiology in these syndromes is the precise handling of cytoplasmic calcium concentration during the excitation-contraction (EC) coupling. One the most important effectors of this EC coupling is the L-type voltage-gated calcium channel Ca_v_1.2 the main isoform expressed in ventricular cardiomyocytes. Its activation leads to an inward calcium current counterbalancing potassium outward current and (1) hence contributes to action potential plateau phase and (2) triggers the calcium-induced calcium-release phenomenon leading to the rapid increase of cytosolic calcium concentration and the contraction of cardiomyocytes^2^. Slight alterations in the calcium concentration during EC coupling could exert a great impact on arrhythmia vulnerability^3^.

Ca_v_1.2 is a multi-subunit complex composed of a pore-forming subunit Ca_v_α_1c,_ and auxiliary subunits including Ca_v_β_2_, and Ca_v_α_2_δ_1_^4^. Ca_v_β_2_ and Ca_v_α_2_δ_1_, encoded by the *CACNB2* and *CACNA2D1* genes, respectively, regulate the gating properties and Ca_v_1.2 trafficking^5–8^. The Ca_v_β_2_ subunit binds to Ca_v_α_1c_ DI-DII loop and promotes the cell surface density of Ca_v_1.2 channels by preventing their degradation by the ubiquitin/proteasome system^9,10^. Co-expression of the Ca_v_α_2_δ auxiliary subunit with Ca_v_β increases the calcium current and hyperpolarises the voltage of Ca_v_1.2 activation^11^. Ca_v_α_1c_, encoded by the *CACNA1C* gene, comprises four transmembrane domains (DI to DIV) connected by cytoplasmic loops and a large intracellular C-terminal domain. The C-terminal part is a major site of regulation of Ca_v_1.2 channel, not only because it is the target site for many regulatory proteins but also due to the auto-inhibition activity of its distal C-terminal part (DCT). *In vivo*, most of the Ca_v_α_1c_ subunit (80%) is cleaved at position 1770, releasing the DCT which acts as an auto-inhibitor peptide on Ca_v_1.2 *via* its non-covalent interaction with the proximal C-terminal (PCT) part^12,13^. Interestingly, despite the fact that the non-cleaved and the cleaved DCT have been shown to promote an auto-inhibitory effect on the calcium current (I_Ca_), Hulme *et al*. showed that the cleaved DCT has a stronger auto-inhibitory effect on I_Ca_ than the non-cleaved one^12^. Knock-in mice expressing Ca_v_α_1c_ with a deleted DCT present perinatal mortality due to heart failure emphasises the fact that the DCT part of L-type calcium channel is primordial for normal cardiac development and function^14,15^. In addition to the loop between DI and DII of Ca_v_α_1c_ which is known to be crucial for the channel inactivation gate, Brunet *et al*. showed that the auto-inhibitory complex could also increase the voltage-dependent inactivation of Ca_v_1.2, further decreasing the calcium influx^16,17^. The non-covalent interaction between the PCT and the DCT leading to the decrease of I_Ca_ has been shown to be calcium-dependent and modulated by calmodulin (CaM)^13^. In presence of low intracellular calcium, the interaction between the PCT and DCT will lead to a decrease of the open probability of Ca_v_1.2. The increase in cytoplasmic calcium will allow the complex Ca^2+^-CaM to interact with the CDD domain localised at the DCT and the IQ and pre-IQ domain from the PCT. This interaction will disrupt the PCT-DCT complex and relieve the auto-inhibition leading to an increase of I_Ca_^13^.

*CACNA1C* was the first gene involved in a multisystem disorder called Timothy syndrome (TS)^18^. Functional analysis of TS variants highlighted two different mechanisms triggering the severe QT interval prolongation and lethal arrhythmia observed in these patients. Missense variants localised at the C-terminal end of DI-S6 induce a nearly complete loss of Ca_v_1.2 voltage-dependent inactivation (VDI) and prolongation of the plateau phase of cardiac action potential while others variants identified in the DIII-DIV loop induced a gain-of-function by increasing the “window” current^19^. Gain-of-function Ca_v_α_1c_ variants were also identified in the DI-DII loop in non-syndromic LQTS patients through an increase in channel surface membrane expression^20^. Furthermore, loss-of-function variants often associated with a shortening of the plateau phase of the action potential in the three Ca_v_1.2 subunits have been reported in patients with BrS with or without short QT^21,22^, ERS^23^, SQTS^24^, and sudden unexplained death in the young (SUDY)^22,25,26^. Different molecular mechanisms were described, such as reduced current density due to impaired channel trafficking for the Ca_v_α_1c_-A39V^21^, activation or inactivation curve shifts for Ca_v_β_2b_-S481L^21^ and SQTS-Ca_v_α2δ1-S755T^24^ or a marked increase in Ca_v_1.2 inactivation rate for Ca_v_β_2b_-T11I^27^. These variants have been associated with phase 2 re-entry^28,29^ or focal activation/re-entry from/within the Purkinje fibres^30^. However, the fact that rare heterozygous missense variants were also present in the healthy populations questioned the implication of variants with experimentally unconfirmed pathogenicity in these diseases^31,32^.

Thus, there is a clear need to explore the function of new Ca_v_1.2 variants to gain a better understanding of the link between these variants and life-threatening ventricular arrhythmias. As a result, we sought to identify new variants of Ca_v_1.2 genes in a cohort of 65 patients, affected by inherited arrhythmia syndrome potentially related to calcium handling and to elucidate their functional consequences.

## Results

We screened the genes encoding the three subunits of Ca_v_1.2, *CACNA1C*, *CACNB2*, and *CACNA2D1* in 65 patients with BrS, SQTS, ERS, IVF or scTdP patients. Among them, 47 (72%) had syncopes or cardiac arrest (43%) and, 21 had a familial history of syncope or SD (32%) (Table 1).

**Table 1:** Clinical characteristics of the patient cohort.

	BrS	SQTS	ERS	IVF	scTdP
Probands (male)	25 (22)	7 (7)	7 (7)	7 (4)	19 (8)
Symptomatic	12 (48%)	3 (42%)	7 (100%)	7 (100%)	19 (100%)
Resuscitated cardiac arrest	3 (12%)	1 (14%)	6 (86%)	7 (100%)	11 (58%)
Mean age at first symptoms (years) ±SD	38.8 ± 14.2	25.3 ± 3.8	33.8 ± 4.3	46.1 ± 12.6	32.9 ± 10.9
Mean age at clinical diagnosis (years) ±SD	45.0 ± 12.4	31.1 ± 5.7	37.8 ± 3.2	49.7 ± 10.6	33.7 ± 10.1
Family history of syncopes or SD	7 (28%)	6 (71%)	1 (14%)	1 (14%)	6 (32%)
Spontaneous BrS type 1 pattern	15 (62.5%)	0	0	0	0
QTc (ms)	364 ± 3	332 ± 3	384 ± 10	409 ± 20	403 ± 31

In addition to frequent polymorphisms (MAF > 1% in all populations), we identified a number of less frequent missense variants of unknown significance (VUS) in the coding sequence of *CACNA1C* in patients affected by BrS, ERS or IVF. No variants were found in *CACNB2* or *CACNA2D1*. We performed functional analyses of missense variants identified in the patients described below.

### Patients and variants

#### Six *CACNA1C* variants were found in 5 patients (7.7%)

**Figure 1:**
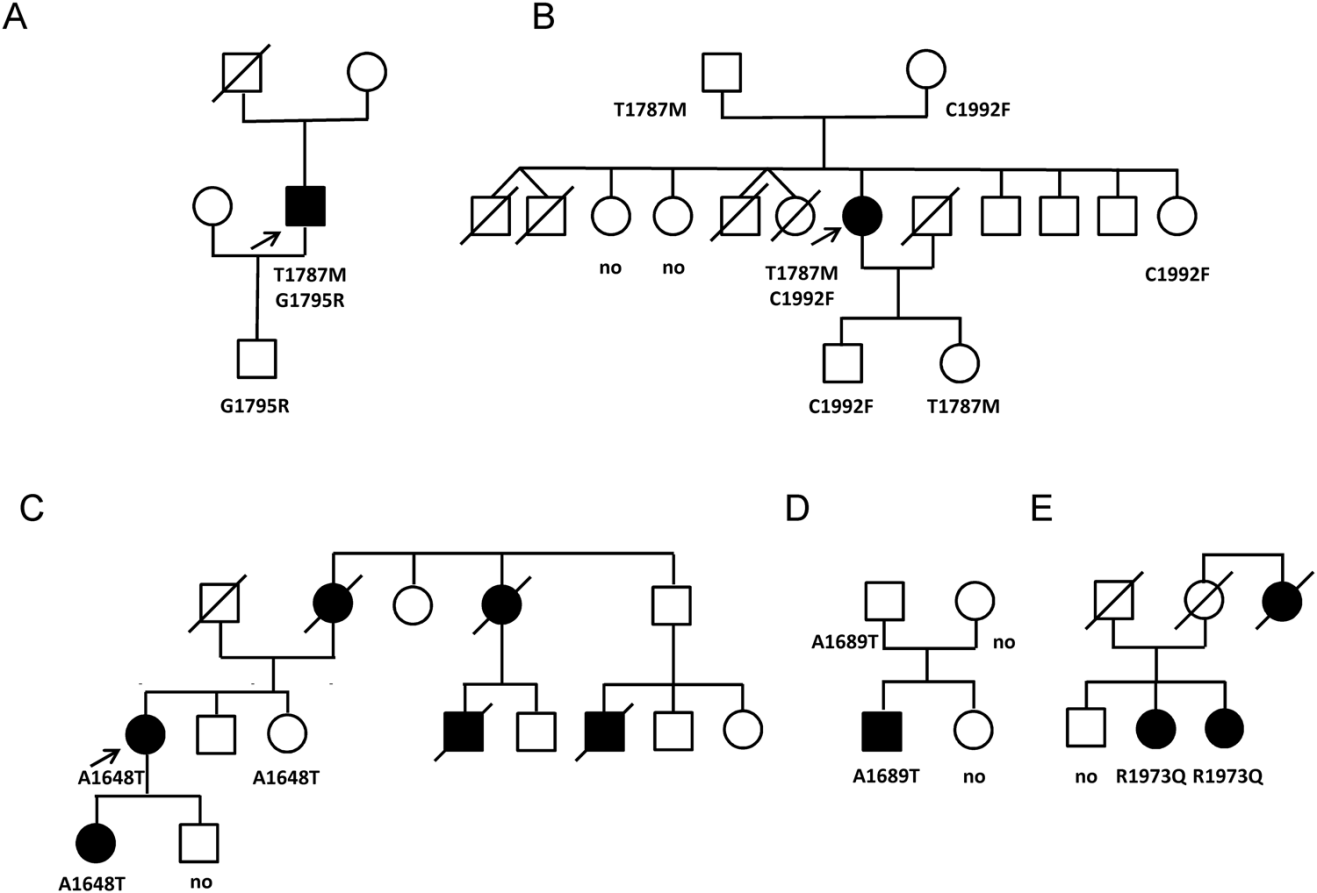
Family pedigrees of Cases 1 to 5 (**A**–**E**) with the members carrying *CACNA1C* variants. The probands are indicated by an arrow. Males are represented by squares, females by circles, affected subjects by filled symbols, and healthy subjects by open symbols. Ca_v_α_1c_ missense variants or their absence (no) are indicated below each subject that has been genotyped.

**Figure 2:**
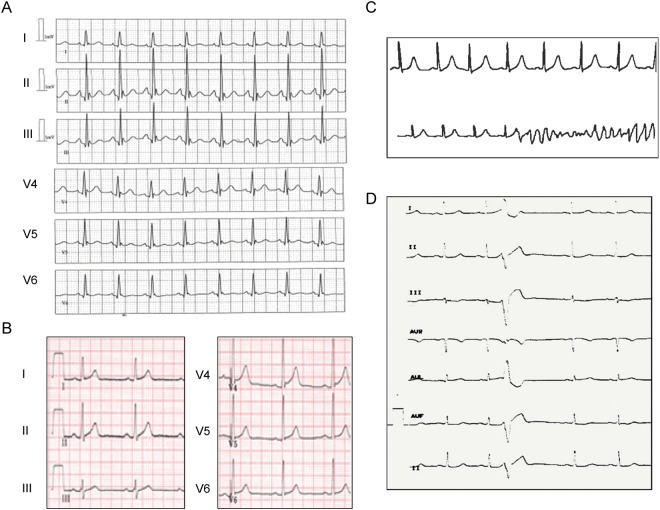
Electrocardiograms of Cases 1 and 2. Case 1, (**A**) just after reanimation showing early repolarisation pattern, (**B**) 5 years later. Case 2, (**C**) short-coupled torsades de pointes, (**D**) short-coupled premature ventricular beats.

Case 1: This man had an aborted SD at the age of 27 when playing soccer (Fig. 1A). All investigations were negative (Holter recording, MRI, coronary angiography, isoprenaline and ajmaline tests). The only aetiology found was an early repolarisation pattern in ECG inferior and lateral leads during the days after resuscitation (Fig. 2A,B). An automatic implantable cardio defibrillator (ICD) was implanted, and the patient experienced only very short sustained episodes of ventricular tachycardia that were not treated by the ICD during the following years. This patient, of Franco-Cameroonian origin, was part of a family with no other cardiac events. He was the only one with an ERS pattern and carrying a missense variant, p.Thr1787Met (Ca_v_α_1c_-T1787M), localised in the C-terminal domain of Ca_v_α_1c_ calcium channel subunit (Fig. 1A and Supplementary Fig. S1C). The threonine residue at position 1787 is conserved through evolution among many species but is replaced by an alanine in horse and dog (Supplementary Fig. S2). The methionine substitution is considered as probably damaging according to Polyphen2 and the DANN Score. Ca_v_α_1c_-T1787M has a minor allele frequency (MAF) of 0.12% according to ExAC database, but it was in fact mostly found in Africans, both in the heterozygous state in 113 of 3565 individuals, and in the homozygous state in one subject (Table 2). As expected, it was absent from our Caucasian control population. Another variant, p.Gly1795Arg (G1795R), with a higher frequency (MAF = 0.54% according to ExAC) was also found in this subject. His healthy son only carried the latter variant, which is frequent in Africans (MAF = 7%), and is therefore considered as likely benign (Supplementary Fig. S1D).

**Table 2:** *CACNA1C* variants.

	Case n°	1 and 2	1 (ERS)	2 (scTdP)	3 (scTdP)	4 (BrS)	5 (scTdP)
	Genomic location Chr12 (GRCh38)	2679712	2679735	2688637	2677207	2677841	2688580
NM_000719	c.DNA variation	c.5360 C > T	c.5383 G > A	c.5975 G > T	c.4942 G > A	c.5065 G > A	c.5918 G > A
	protein variation	T1787M	G1795R	C1992F	A1648T	A1689T	R1973Q
NM_199460	c.DNA variation	c.5504 C > T	c.5527 G > A	c.6224 G > T	c.5086 G > A	c.5209 G > A	c.6167 G > A
	protein variation	T1835M	G1843R	C2075F	A1696T	A1737T	R2056Q
MAF	ExAC (All) (%)	0.12	0.54	0.01	0.008	0.0031	0.36
	ExAC (Africans) (%)	1.61	7.03	0.05	0	0	0.02
	ISB Kaviar 3 (%)	0.12	0.39	0.008	0.006	0.0026	0.31
Pathogenicity	Polyphen 2	Probably damaging: 0.993	Benign: 0.001	Probably damaging: 0.998	Possibly damaging: 0.948	Possibly damaging: 0.949	Probably damaging: 0.977
	Mutation Taster	Polymorphism	Polymorphism	Disease causing	Disease causing	Disease causing	Disease causing
	ClinVar	Benign- Likely benign	Benign- Likely benign	Uncertain significance	Uncertain significance	Uncertain significance	Conflicting interpretations of pathogenicity
	DANN Score	0.9966	0.9892	0.9872	0.8767	0.9982	0.8254

Case 2: This woman had syncope at rest at the age of 43 years, followed by a resuscitated cardiac arrest during hospitalisation with the detection of scTdP originating from Purkinje fibres (Fig. 2C). The origins of her family are from La Reunion, where four SD of unknown aetiology occurred in young children (Fig. 1B). A coronary angiography and echocardiography excluded ischaemic or structural abnormalities. She received an ICD and a verapamil treatment of 240 mg twice a day. A right ventricular scintigraphy performed at the age of 49 did not reveal any signs of arrhythmogenic right ventricular cardiomyopathy (ARVC). During the follow-up period, several asymptomatic non-sustained ventricular tachycardia events were recorded by the ICD, beginning with premature ventricular beats (PVB) with short coupling intervals (320 ms) (Fig. 2D). When she skipped her verapamil treatment, non-sustained ventricular tachycardia with a feeling of faintness and palpitation was also recorded. Electrocardiographic screening of the other family members did not show any abnormalities.

The proband carried two variants in *CACNA1C*, Ca_v_α_1c_-T1787M and p.Cys1992Phe (C1992F) (Fig. 1B, Supplementary Fig. S1C,F). The first variant, Ca_v_α_1c_-T1787M, was the same as in the previous family. The second *CACNA1C* variant, C1992F, was found in East Asians (7/8536 alleles) and Africans (5/8536 alleles) with an overall MAF of 0.01% in ExAC. The cysteine residue at position 1992 in Ca_v_α_1c_ is highly conserved among species (Supplementary Fig. S2) and its substitution with a phenylalanine is considered as probably damaging according to Polyphen2, Mutation Taster and the DANN Score (Table 2). This variant is also located in the C-terminal region of the channel subunit. By genotyping some family members, we found that the proband was the only carrier of two variants in Ca_v_1.2 calcium channel subunits; other family members who have been genotyped were only carriers of one of these two variants.

Three additional VUS were identified in the proband: p.Leu618Phe (L618F) in the cardiac sodium channel (*SCN5A*, NM_198056, c.1852C > T) with an overall MAF of 0.057%, and two variants in desmosomal proteins known to be associated with ARVC: p.Asn1865Trp (N1865W) in desmoplakin (*DSP*, NM_004415.2, c.5593 A > T), with a frequency of 0.051%, and p.Val842Ile (V842I) in plakophilin 2 (*PKP2*, NM_004572, c.2524 G > A), with an MAF of 0.0049% (Supplementary Fig. S3).

Case 3: This patient comes from a French family where several SD occurred in young adults (Fig. 1C). She had a resuscitated cardiac arrest after lunch and her ECG showed scTdP, possibly originating from Purkinje tissue. Her own mother had died at the age of 25 during the night. Her daughter also had ventricular fibrillation originating from the Purkinje network and numerous scPVB episodes at the age of 15 years. An ICD was implanted and catheter ablation performed. A *CACNA1C* missense variant, p.Ala1648Thr (A1648T), was detected in the affected mother and daughter (Fig. 1C and Supplementary Fig. 1A). The alanine residue at position 1648 is located in a well conserved region of the protein (Supplementary Fig. S2) and its substitution with a threonine was considered as possibly damaging according to several prediction tools (Table 2). The variant was only found in 10 Europeans (10/70496 alleles) with an overall frequency of 0.01% according to the ExAC database.

Case 4: This French patient presented an aborted SD at the age of 21 during exercise, without any aetiology. Echocardiography and cardiac MRI did not show any morphological abnormality, and a stress test did not show any argument in favour of a catecholaminergic tachycardia. His basal ECG was normal and ajmaline test was negative. The patient was implanted with an ICD without other medical treatment. During the follow-up, the ICD delivered several appropriate shocks on ventricular tachycardia. At the age of 26 years, a second ajmaline test was performed and was positive. There was no family history of syncope or cardiomyopathy. A *CACNA1C* variant was identified, p.Ala1689Thr (A1689T), affecting an alanine that is well conserved during evolution (Fig. 1D, Supplementary Figs S1B and S2). This variant was absent from our Caucasian control population and considered as possibly damaging according to Polyphen2, Mutation Taster and the DANN Score (Table 2).

Case 5: Two sisters originating from La Reunion Island were diagnosed with IVF associated with scTdP. Their great aunt, who had episodes of syncope, died suddenly at age 50. The first sister had two consecutive syncopal episodes and resuscitated cardiac arrest at age 39 with scTDP (300 msec). Subsequently, she was implanted with an ICD. She experienced an appropriate shock one year after implantation and presented an electrical storm three years after. Her sister had a first syncope during fever at 36 and then later complained of palpitations. She presented scPVB (320 msec) at ECG and scTdP (260 msec) was detected on Holter recording during palpitations. She was implanted with an ICD at age 45, but never experienced appropriate shocks. The two sisters were treated with verapamil. They both carry the same *CACNA1C* variant, p.Arg1973Gln (R1973Q) (Fig. 1E and Supplementary Fig. S1E). The arginine residue at position 1973 of Ca_v_α_1c_ is highly conserved among species (Supplementary Fig. S2) and is located within the Ca_v_α_1c_ C-terminal domain. The glutamine substitution was considered as probably pathogenic according to Polyphen2, Mutation Taster and the DANN Score. However, this variant was present in all ethnic populations in the ExAC database with an overall MAF of 0.36% (Table 2).

### Loss-of-function of the Ca_v_α_1c_-T1787M variant in *CACNA1C*

**Figure 3:**
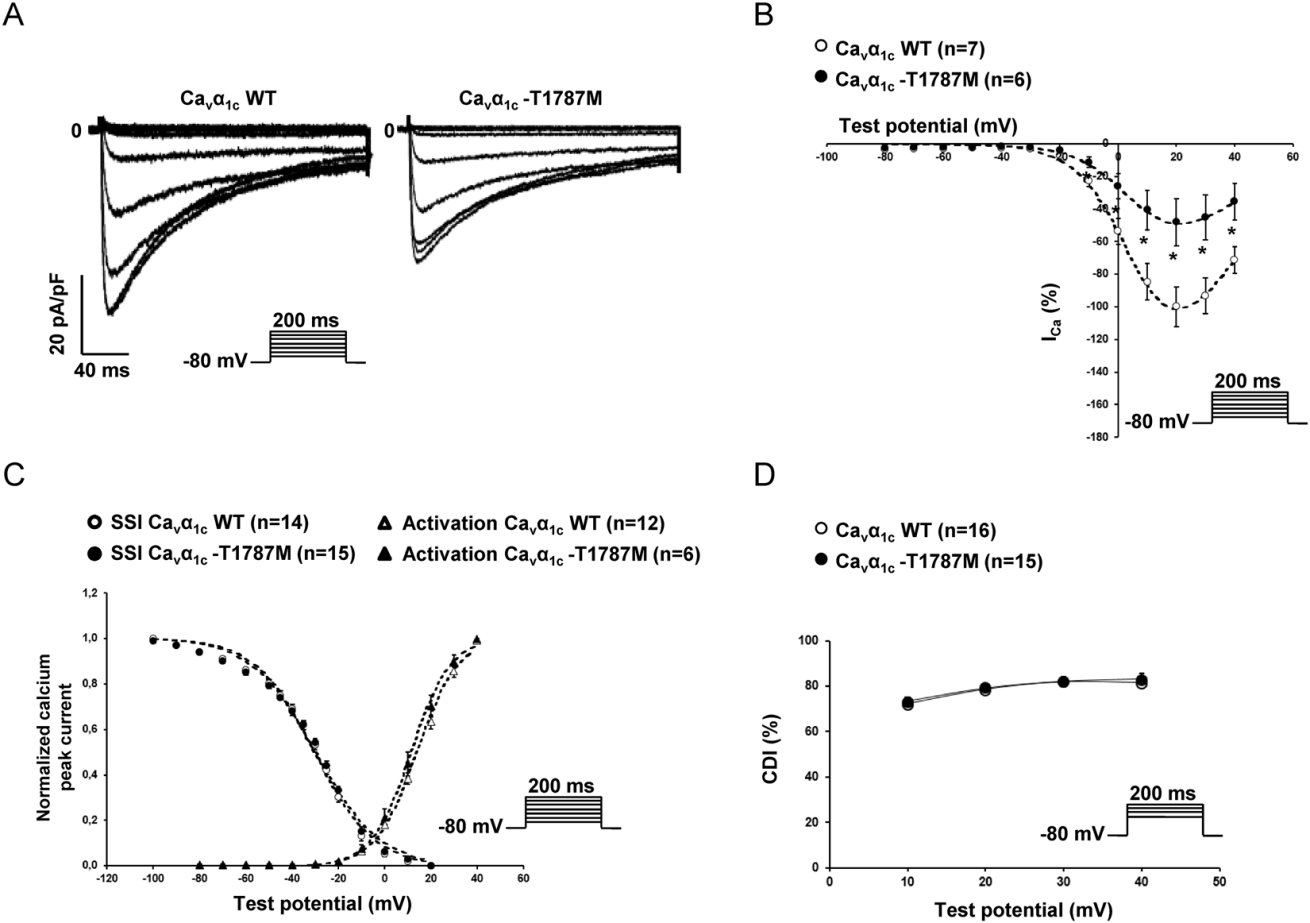
Loss-of-function of Ca_v_α_1c_-T1787M variant: calcium currents (I_Ca_). (**A**) Representative whole-cell I_Ca_ traces showing the decrease of current density with the Ca_v_α_1c_-T1787M variant compare to WT. (**B**) Current-voltage relationships in cells transfected with either WT (○) or Ca_v_α_1c_-T1787M variants (●) of calcium channels (*p < 0.05). (**C**) Steady-state inactivation and activation curves of either WT (white symbol) or Ca_v_α_1c_-T1787M variants (back symbol) of calcium channels showing no major alterations. (**D**) Calcium-dependent inactivation (CDI) with either WT (○) or Ca_v_α_1c_-T1787M variants (●) of calcium channels showing no alterations. The number of cells is indicated in parentheses.

**Figure 4:**
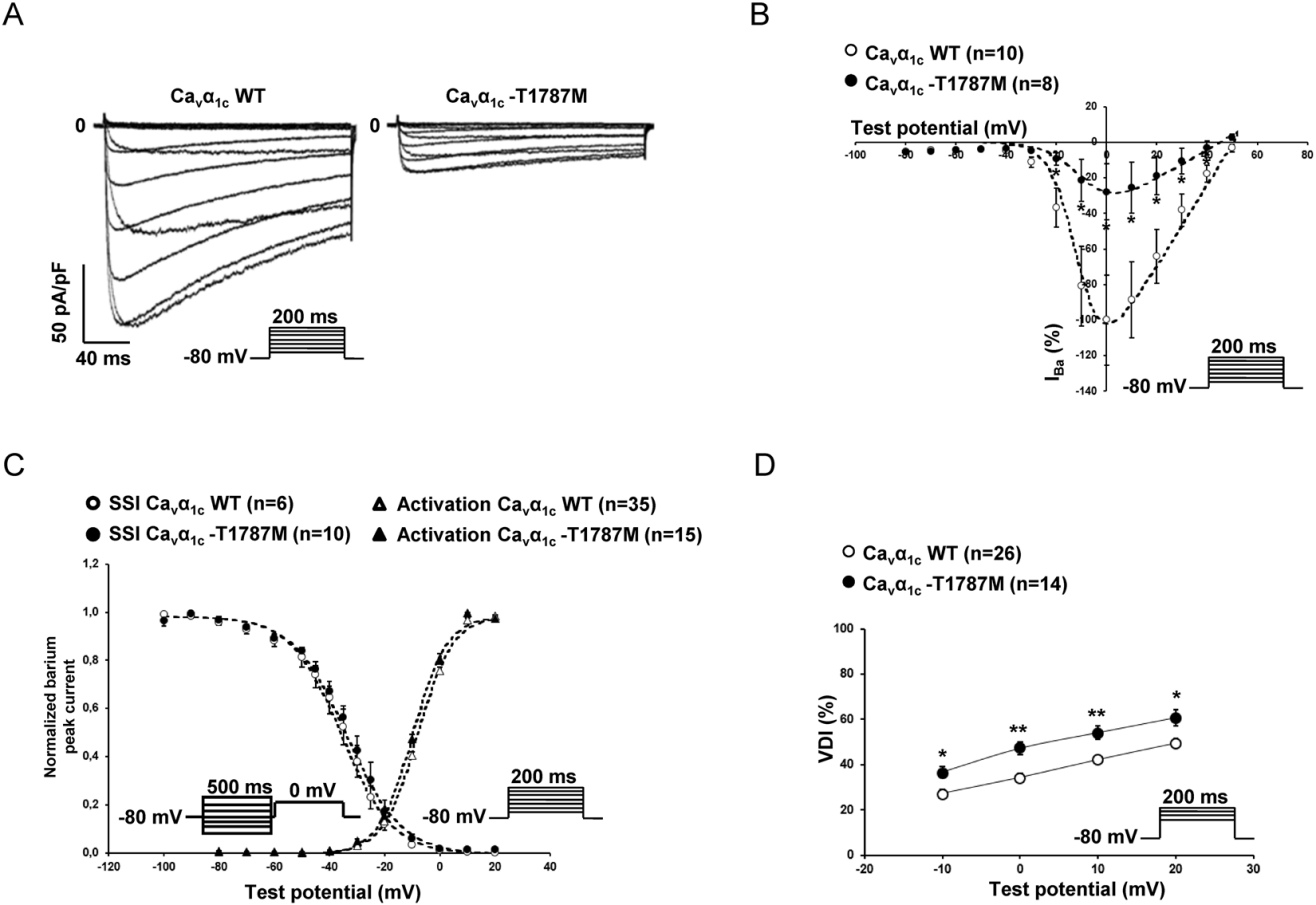
Loss-of function of Ca_v_α_1c_-T1787M variant: barium currents (I_Ba_). (**A**) Representative whole-cell I_Ba_ traces showing the decrease of current density with the Ca_v_α_1c_-T1787M variant compare to WT. (**B**) Current-voltage relationships in cells transfected with either WT (○) or Ca_v_α_1c_-T1787M variants (●) of calcium channels (*p < 0.05). (**C**) Steady-state inactivation and activation curves of either WT (white symbol) or Ca_v_α_1c_-T1787M variant (back symbol) of calcium channels showing no major alterations. (**D**) Voltage-dependent inactivation (VDI) with either WT (○) or Ca_v_α_1c_-T1787M variant (●) of calcium channels showing an increase of the VDI (*p < 0.05 and **p < 0.01). The number of cells is indicated in parentheses.

The six C-terminal variants, A1648T, A1689T, T1787M, G1795R, R1973Q, and C1992F were introduced by mutagenesis in the Ca_v_α_1c_ subunit. Functional patch-clamp studies showed that, despite a slight shift in steady-state inactivation for the variants A1648T and A1689T in the presence of extracellular calcium, only the Ca_v_α_1c_-T1787M variant leads to a decrease in I_Ca_ and barium current (I_Ba_) compared to the wild-type (WT). No major alteration of the main biophysical properties was found for Ca_v_α_1c_-T1787M (steady-state activation and inactivation) (Figs 3–4, Supplementary Figs S4–S9, Tables 3 and 4). Immunoblotting studies showed no alterations in calcium channel subunit expression after the over-expression of Ca_v_α_1c_-T1787M (Supplementary Fig. S10A). Moreover, biotinylation of cell surface proteins showed that the surface expression of WT or Ca_v_α_1c_-T1787M was not significantly different (113 ± 11 vs 94 ± 17%, respectively, n = 3 experiments) (Supplementary Fig. S10B).

**Table 3:** Biophysical parameters of Ca_v_1.2 variants with calcium.

	Normalized maximum peak current	Activation		Steady-state inactivation	
	%	V_1/2_ (mV)	K	V_1/2_ (mV)	K
WT	100 ± 18 (19)	15.45 ± 1.64 (19)	7.92 ± 0.73 (19)	−30.43 ± 1.05 (15)	12.96 ± 0.79 (15)
A1648T	128 ± 15 (5)	15.45 ± 2.29 (5)	8.41 ± 0.24 (5)	−34.40 ± 1.56 (5)*	14.73 ± 0.70 (5)
A1689T	112 ± 46 (7)	12.84 ± 1.31 (7)	8.01 ± 0.22 (7)	−24.11 ± 0.69 (7)*	13.61 ± 0.65 (7)
T1787M	48 ± 14 (6)*	11.86 ± 1.89 (6)	8.19 ± 0.31 (6)	−29.90 ± 1.04 (15)	13.73 ± 0.67 (15)
G1795R	94 ± 54 (5)	11.68 ± 1.80 (5)	8.23 ± 0.63 (5)	−25.73 ± 1.71 (7)	14.33 ± 0.67 (7)
R1973Q	135 ± 28 (6)	12.29 ± 0.97 (7)	7.87 ± 0.13 (7)	−25.61 ± 1.29 (9)	13.21 ± 0.86 (9)
C1992F	109 ± 30 (6)	12.21 ± 1.04 (7)	7.94 ± 0.18 (7)	−25.84 ± 1.64 (6)	12.69 ± 0.57 (6)

Activation and steady-state inactivation parameters (see material and methods) of WT and variants of Ca_v_1.2 channels. In bold, values significantly different from WT (*p < 0.05). The number of cells is indicated in parentheses.

**Table 4:** Biophysical parameters of Ca_v_1.2 variants with barium.

	Normalized maximum peak current	Activation		Steady-state inactivation	
	%	V_1/2_ (mV)	K	V_1/2_ (mV)	K
WT	100 ± 10 (46)	−7.49 ± 0.42 (35)	6.18 ± 0.13 (35)	−35.74 ± 1.01 (43)	12.24 ± 0.96 (43)
A1648T	92 ± 20 (10)	−6.53 ± 0.73 (11)	5.83 ± 0.20 (11)	−37.93 ± 1.45 (9)	11.68 ± 0.24 (9)
A1689T	94 ± 25 (10)	−9.14 ± 0.80 (5)	6.06 ± 0.11 (5)	−33.67 ± 1.20 (6)	7.54 ± 0.40 (6)
T1787M	28 ± 16 (8)*	−5.53 ± 1.63 (15)	5.84 ± 0.45 (15)	−32.93 ± 2.50 (10)	9.18 ± 0.80 (10)
G1795R	93 ± 7 (8)	−6.35 ± 0.90 (10)	6.08 ± 0.18 (10)	−37.35 ± 1.59 (10)	12.43 ± 0.63 (10)
R1973Q	90 ± 14 (20)	−7.31 ± 0.46 (18)	6.09 ± 0.13 (18)	−37.60 ± 0.76 (19)	11.77 ± 0.31 (19)
C1992F	97 ± 12 (19)	−6.23 ± 0.73 (17)	5.93 ± 0.12 (17)	−35.70 ± 0.74 (16)	12.67 ± 0.39 (16)
A1770*	100 ± 10 (21)	−12.29 ± 0.75 (20)	5.61 ± 0.16 (20)	−38.77 ± 1.13 (21)	9.96 ± 0.42 (21)
A1770*/WT	64 ± 9 (24)	−9.30 ± 0.70 (24)	5.94 ± 0.16 (24)	−39.31 ± 0.72 (24)	10.32 ± 0.41 (24)
A1770*/T1787M	39 ± 6 (23)*	−9.39 ± 0.88 23)	6.18 ± 0.18 (23)	−38.61 ± 1.22 (23)	10.95 ± 0.47 (23)

Activation and steady-state inactivation parameters (see material and methods) of WT, variants, and truncated constructs of Ca_v_1.2 channel. In bold: WT vs Ca_v_α_1c_-T1787M and underlined A1770*/WT vs A1770*/T1787M (*p < 0.05). The number of cells is indicated in parentheses.

Interestingly, while the calcium-dependent inactivation (CDI) was not altered by the different variants (Fig. 3D, Supplementary Fig. S11), the voltage-dependent inactivation (VDI) was significantly increased in the presence of the Ca_v_α_1c_-T1787M variant only (Fig. 4D, Supplementary Fig. S12).

### The inhibition mediated by the cleaved CT part on the CT-cleaved Ca_v_α_1c_ construct is stronger when the Ca_v_α_1c_-T1787M variant is present

**Figure 5:**
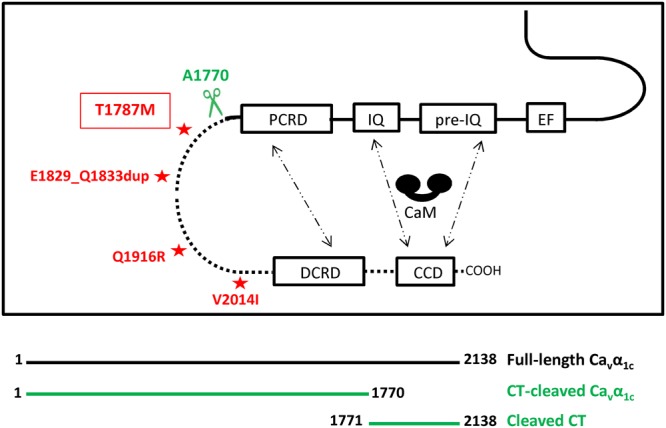
Scheme of the C-terminal part of the voltage-gated calcium channel. Schematic representation modified from Lyu *et al*. 2017 showing the C-terminal domain of Ca_v_α_1c_ with EF-hand like region (EF), pre-IQ, IQ, PCRD, DCRD, and CaM-competitive domain (CCD)^62^. The interaction between the PCRD and the DCRD forming the auto-inhibitory complex and the different sites of interaction with CaM (pre-IQ, IQ, and CCD) are depicted with dashed arrows. Asterisks correspond to human loss-of-function variants (present data, red)^22^. The *in vivo* cleavage site by proteolysis correspond to 1764-NANINNANN-1772 in human. The scissors show the position of the truncation of Ca_v_α_1c_ subunit performed in this study (A1770, 1800 in rabbit).

Since no modification of the main biophysical properties of the Ca_v_α_1c_-T1787M and a normal expression at the membrane were found, another mechanism should explain the observed reduced current density. Interestingly, the variant is localised close to the *in vivo* proteolytic cleavage site (A1770) at the beginning of the cleaved C-terminal (cleaved CT) involved in the auto-inhibition process of Ca_v_1.2 channel (Fig. 5). The auto-inhibition mechanism is mediated by the interaction of the proximal C-terminal regulatory domain (PCRD) localised in the PCT with the distal C-terminal regulatory domain (DCRD) present in the DCT (Fig. 5). This interaction has also been shown to be modulated by CaM *via* its interaction with the CaM-competitive domain (CCD) and the pre-IQ and IQ motif (see introduction) (Fig. 5).

**Figure 6:**
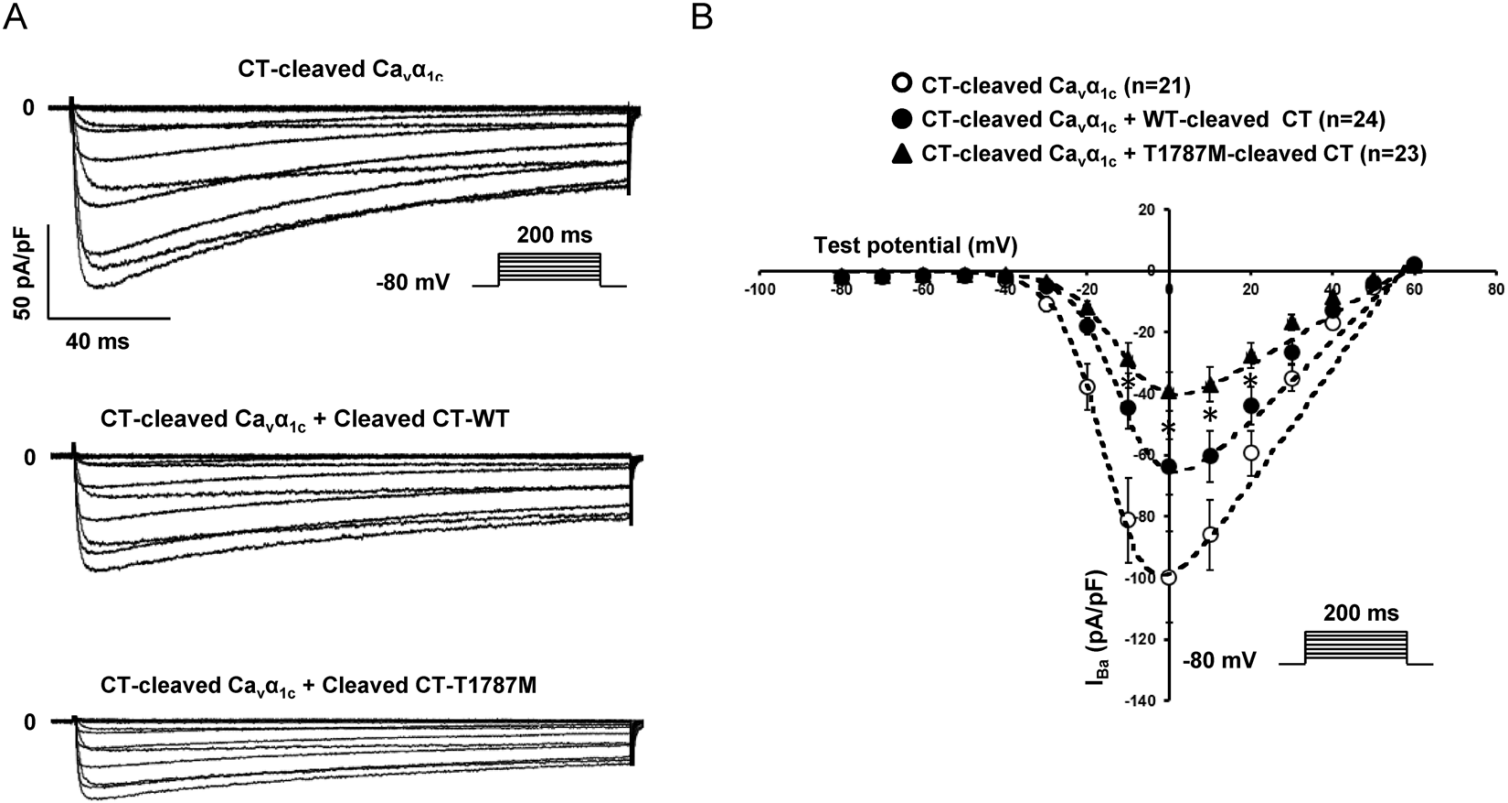
The I_Ba_ inhibition mediated by the cleaved CT part on the CT-cleaved Ca_v_α_1c_ construct is stronger when the Ca_v_α_1c_-T1787M variant is present. (**A**) Representative raw traces of I_Ba_ density recorded in presence of CT-cleaved Ca_v_α_1c_ alone or with either WT or T1787M-cleaved CT part. (**B**) I-V relationships showing the effect mediated by T1787M variant on the CT-cleaved Ca_v_α_1c_ when introduced in the cleaved CT part (*p < 0.05: CT-cleaved Ca_v_α_1c_ + WT-cleaved CT *vs* CT-cleaved Ca_v_α_1c_ + T1787M-cleaved CT). The number of cells is indicated in parentheses.

To investigate whether Ca_v_α_1c_-T1787M could modulate this inhibition, a truncated construct at the *in-vivo* cleavage site has been generated (A1770*) (Fig. 5). As already published, the current produced by the A1770* truncated form is greater than that of the WT channel (WT: 34 ± 4 pA/pF (n = 36) and A1770*: 100 ± 15 pA/pF (n = 21); p < 0.05)^12^. Barium currents mediated by this CT-cleaved Ca_v_α_1c_ form were decreased by 36 ± 9% when co-expressed with the WT-cleaved CT part with a significant shift of the steady-state activation curve (Fig. 6 and Table 3). However, when co-expressed with the cleaved CT part harbouring the Ca_v_α_1c_-T1787M variant, I_Ba_ was further decreased without biophysical property alterations compared to the WT-cleaved CT, suggesting that the presence of the variant increases the auto inhibition effect (−38 ± 10%, CT-cleaved Ca_v_α_1c_ + WT-cleaved CT vs CT-cleaved Ca_v_α_1c_ + T1787M-cleaved CT; p < 0.05) (Fig. 6 and Table 4).

## Discussion

Calcium influx *via* the Ca_v_1.2 L-type calcium channel provides a multi-functional signal that triggers muscle contraction, controls action potential and regulates gene expression^33^. Therefore, a dysregulation of the calcium channel function may lead to cardiac rhythm disorders. A limited number of Ca_v_1.2 variants have been studied; thus, the extent of their potential role remains unknown and requires validation by functional studies^19^. Some mutations in the *CACNA1C* gene were first identified in Timothy syndrome, causing extreme QT prolongation and SD^18^. The role of these gain-of-function mutations of Ca_v_1.2 in ventricular repolarisation prolongation was confirmed by the discovery of several other mutations in non-syndromic sporadic and familial LQTS cases^20,34^. On the other hand, several missense variants in the Ca_v_1.2 subunits were reported as loss-of-function mutations in BrS, SQTS, ERS and SUDY patients^21,22,24–26^. The low penetrance of these variants suggests a complex genetic inheritance with an accumulation of several genetic risk factors of variable frequencies^35^.

In this study, we aimed to identify variants in the three genes encoding Ca_v_1.2 subunits in patients affected with various kinds of arrhythmia disorders (BrS, SQTS, ERS, IVF and scTdP). We reported six missense variants in *CACNA1C*, one in a BrS patient, and the five others in patients with ERS and IVF. Our patch-clamp experiments revealed that, among the variants found in this study, the Ca_v_α_1c_-T1787M variant was the only Ca_v_α_1c_ variant to cause a significant reduction in I_Ca_ and I_Ba_. Barium current recordings also showed an increase in VDI for the Ca_v_α_1c_-T1787M variant compared to WT. Since I_Ca_ is involved in the cardiac action potential plateau phase, an increase in Ca_v_1.2 VDI combined with a decrease in I_Ca_ would lead to a shorter plateau phase and thus a shorter action potential. Interestingly, the QTc intervals of Cases 1 and 2 were relatively short (358 ms and 380 ms, respectively). Several other mutations (E1829_Q1833dup, Q1916R, V2014I) linked to BrS or ERS patients have been reported in this region, flanked by the PCRD and the DCRD, also leading to a loss-of-function (Fig. 5)^21–23^. The E1829_Q1833dup, close to the Ca_v_α_1c_-T1787M variant, induced a drastic decrease in the calcium current amplitude. Unfortunately, the molecular mechanism of this loss-of-function is not known.

The C-terminal part of Ca_v_α_1c_ subunit serves as a scaffold for the targeting and localisation of signalling molecules including calmodulin^36^, calmodulin-dependent protein kinase II^37^, PKA and its anchoring proteins AKAP15^38^. In addition, this C-terminal domain has also been shown to be cleaved *in vivo*^12^.This mechanism will allow the DCRD, from the cleaved CT, to non-covalently bind to the PCRD of the CT-cleaved Ca_v_α_1c_ in order to form a complex^12^. Finally, this complex has been shown to decrease the open probability and modulate the coupled channel gating of Ca_v_1.2 channel leading to a global decrease of I_Ca_^12,39,40^.

Interestingly, experiments performed in this study using the CT-cleaved Ca_v_α_1c_ construct suggest a potential role for the Ca_v_α_1c_-T1787M variant in the auto-inhibitory effect. The fact that the decrease in I_Ba_ observed in presence of WT-cleaved CT is amplified when the mutation is introduced in this cleaved CT suggests that the Ca_v_α_1c_-T1787M variant could potentially increase the interactions between the PCRD and the DCRD. Stronger interaction such as this could lead to an absence of gating coupling between Ca_v_1.2 channels, as proposed by Navedo and co-workers, and/or a greater inhibition of the barium current due to the auto-inhibitory mechanism suggested by Wei and colleagues^39,40^. Nevertheless, further experiments should be performed to challenge these hypotheses.

The fact that only the V_1/2_ for the steady-state inactivation of the A1648T and A1689T variants were significantly shifted in the presence of extracellular calcium is surprising and could be due to an artefact of the analysis. In fact, the sigmoid curves calculated by the software do not always fit with the raw data points recorded. Based on these observations, and due to the fact that neither the peak current densities nor other biophysical parameters are altered, additional experiments are required to confirm these alterations.

Although we identified this pathogenic variant in two IVF patients, we did not find pathogenic variants responsible for BrS or SQTS in our cohort as previously reported, and two of the three variants identified in scTdP appeared to be non-functional polymorphisms. We identified six variants in *CACNA1C*, two of which, R1973Q and Ca_v_α_1c_-T1787M, have already been reported by Burashnikov *et al*. in two BrS patients and one BrS patient, respectively^22^. The latter did not form part of those who underwent functional analyses^22^. Measures of evolutionary sequence conservation and allele frequency are widely used as indicators of the deleteriousness of protein variation but their predictive power is limited by statistical and biological factors^41^. Interestingly, among the identified variants, the five most conserved residues in Ca_v_1.2 Ca_v_α_1c_ were non-pathogenic and our only pathogenic variant, Ca_v_α_1c_-T1787M, was not perfectly conserved among all mammals and was found in 0.8% of Africans. Many deleterious variants do not show a strong conservation signature and conversely, strong conservation does not necessarily mean that the variant has an effect on disease risk, which is consistent with our results. Due to the relatively high frequency in Africans, the Ca_v_α_1c_-T1787M variant would initially have been excluded during exome sequencing data analysis according to classical criteria. Since it was found in two unrelated patients with ventricular fibrillation: Cases 1 and 2 originating from Cameroon and from La Reunion, an island of known mixed origin, we performed functional analysis. A strong association between early repolarisation pattern and/or sudden death was reported in several studies including Cameroonian patients^42–44^. As seen for a number of other ECG-derived traits, such as QT interval, an early repolarisation pattern is a heritable component^45^.

The results presented in this study suggest that the *CACNA1C* variant Ca_v_α_1c_-T1787M is likely a potential new risk factor contributing to the development of ventricular arrhythmogenicity in Africans. The best-known African pro-arrhythmic variant is Na_v_1.5-S1103Y, which has a MAF of 13% in African Americans^46–48^. The Y1103 allele carriers have an inherent susceptibility to potentially lethal arrhythmias, and are frequent in cohorts of patients with SD^49^ and Sudden Infant Death Syndrome^48,50^. Additional factors, such as other genetic variants^51^, medications^46^, heart failure associated with a reduced ejection fraction^52^, and hypokalemia associated with diuretic use^53^ can contribute to fatal arrhythmias. Interestingly, case 1 is also a heterozygous carrier of the Na_v_1.5-S1103Y variant. The association of the two variants Ca_v_α_1c_-T1787M and Na_v_1.5-S1103Y might contribute to the patient phenotype.

In addition to the Ca_v_α_1c_-T1787M, Case 2 inherited a variant in the sodium channel, L618F, and two variants in desmosomal proteins, N1865Y in desmoplakin and V842I in plakophilin 2 from her mother. The leucine residue at position 618 is located in the I-II linker domain of the cardiac Na^+^ channel, next to L619F mutation, reported in a LQT3 child and associated with a defective inactivation, a faster recovery and an increase in window current^54^. These two adjacent leucines are conserved in four other sodium channels (Na_v_1.1, Na_v_1.2, Na_v_1.6, Na_v_1.7), and the variant L618F is mostly found in Africans (MAF = 0.64%). A previous study reported no change in window current and late sodium current for L618F in the background of the hH1-Q1077 isoform^55^. Nevertheless, since variant alterations may vary according to the isoform used in expression studies, we studied this variant again in the most abundant hH1a-Q1077del Na_v_1.5 isoform background^47,56^ and confirmed the benign effect of this variant (Supplementary Fig. S13). Accordingly, no QT prolongation was observed in the family members carrying this variant. As for the desmosomal protein variants, PKP2-V842I and DSP-N1865Y, these rare variants are both of unknown significance. They have never been reported and were found in 6/60,000 and 1/60,000 subjects in the ExAC database. MRI did not diagnose any structural abnormalities in Case 2, nor in her sisters carrying one or two of these variants. Only the mother at the age of 72 showed a mild dilatation of the right ventricle. Interestingly, some PKP2 variants have been reported to locally modify sodium channel density at intercalated disks^57^, and the silencing of desmoplakin decreases connexin 43/Na_v_1.5 expression and sodium current in HL1 cardiac muscle cell line^58^. The accumulation of all of these variants in a same subject may contribute to the ventricular hyperexcitability observed in Case 2.

Identifying the origin of cardiac channelopathies in patients with rare arrhythmias provides a unique opportunity to identify at risk surviving relatives and the possible prevention of future SD. This study underlined the hypothesis of a combinatory effect of several variants to explain the phenotype of the patients, especially Cases 1 and 2. Indeed, the Ca_v_α_1c_-T1787M variant may not be sufficient to cause the phenotype observed in patients, but like Na_v_1.5-S1103Y, it could play a role combined with additional risk factors.

## Conclusions

Overall, this study shows that the Ca_v_α_1c_-T1787M variant present in 0.8% of the African population was identified in two out of 65 patients (3.1%) with resuscitated cardiac arrest. This variant causes a potentially arrhythmogenic Ca_v_1.2 loss-of-function.

### Limitations of the study

Patch-clamp analysis of five of our variants did not show any abnormal Ca_v_1.2 activities. Nevertheless, based on the limitations due to the experimental model used in this investigation, we cannot exclude that those variants could lead to abnormal calcium handling in native cardiomyocytes. In fact, in cardiac cells, contrary to the TsA-201 cell line, Ca_v_1.2 channels are mainly expressed in the T-tubule structure within macro-molecular complexes allowing specific regulation of the voltage-gated calcium channel^59^. Further investigations using another model close to cardiomyocytes such as cardiomyocytes derived from induced pluripotent stem (iPS) cells from the patients could be an alternative to decipher whether those variants are pathogenic or not.

## Material and Methods

### Patients

The patient cohort consisted of probands originating from different countries with BrS, SQTS, ERS, IVF and scTdP according to the international consensus criteria^1,60^. Underlying structural heart disease was excluded by echocardiography. Laboratory tests excluded acute ischaemia and metabolic or electrolyte disturbances.

Symptomatic and asymptomatic BrS probands, displayed a BrS type-1 pattern on electrocardiogram (ECG) (ST segment elevation ≥2 mm in one or more right precordial leads), either spontaneously, or induced by a sodium blocker challenge test (ajmaline or flecainide). All were negative for mutations in *SCN5A*, *SCN1B*, *SCN2B*, *SCN3B*, *KCNJ8*, *KCNE3*, *KCND3*, and *RANGRF*.

SQTS probands had either QTc intervals inferior to 330 msec, or QTc between 330 and 360 msec with a family history of SD before the age of 40, or survival of VT/VF episode in absence of heart disease.

ERS was diagnosed in the presence of J-point elevation ≥1 mm in ≥2 contiguous inferior and/or lateral leads of a standard 12-lead ECG in patients with or without cardiac arrest from otherwise unexplained VF/polymorphic VT.

Twenty-six patients had syncopes with a normal ECG at rest and during exercise stress test. Seven of them had a resuscitated cardiac arrest and were diagnosed as IVF in absence of cardiac, respiratory, metabolic and toxicological aetiologies. Nineteen presented with scPVB or scTdP originating from Purkinje fibres confirmed by electrophysiological study and were classified as scTdP.

Blood samples were obtained after signed informed consent forms were collected for genetic analyses and upon approval of the local ethics committee of the Saint-Louis Hospital. The study was conducted according to the principles of the Helsinki Declaration.

### CACNA1C, CACNB2 and CACNA2D1 analysis

Genomic DNA was extracted from peripheral blood leukocytes according to standard procedures. The genes *CACNA1C (*NM_199460.3), *CACNB2 (*NM_000724.3), and *CACNA2D1* (NM_000722.3) were first screened in 2012 in 47 patients using a high-resolution melt (HRM) analysis method in a real-time PCR thermocycler (LightCycler 480^®^, Roche Diagnostics^®^). Primers were designed according to HRM specifications and permitted the amplification of all exons and splice junctions (available upon request). Results were analysed using the gene-scanning module of the LightCycler 480^®^ software (Roche Diagnostics^®^). When a new variant was identified, the PCR product was purified and sequenced with the Big Dye Terminator v.3.1 kit (Applied Biosystems^®^). Sequencing was performed on the ABI PRISM 3730 automatic DNA sequencer (Applied Biosystems^®^). Variants were identified by visual inspection of the sequence with Codon Code Aligner^®^ software (4.1.1 version). Then, the frequency of novel non-synonymous variants was evaluated by screening 300 Caucasian and 100 North African unrelated healthy controls.

In addition, a whole exome sequencing (WES) was performed in 2015 by IntegraGen (Evry, France) for 18 probands presenting IVF associated with scPVB or scTdP. Genomic DNA were fragmented by sonication and purified to yield fragments of 150–200 bp. Paired-end adaptor oligonucleotides from the NEBNext Direct kit (New England Biolabs) were ligated on repaired, tailed fragments and then purified and enriched by 8 PCR cycles. From these purified libraries, 1200 ng were then hybridised to the SureSelect oligo probe capture library (SureSelect XT Clinical Research Exome −54 Mb, Agilent) for 72 hours. After hybridisation, washing, and elution, the eluted fraction was PCR-amplified with 9 cycles, purified and quantified by QPCR to obtain sufficient DNA template for downstream applications. Each eluted-enriched DNA sample was then sequenced on an Illumina HiSeq4000 as paired-end 75b reads. Bioinformatic analyses were performed by IntegraGen. Reads were aligned with human genome assembly GRch37. The variants were filtered using IntegraGen ERIS platform.

The prediction of the amino acid substitution effect on protein structure and function was assessed using different prediction tools including Polyphen2, Mutation Taster, ClinVar and the DANN Score^61^. The variant allele frequencies in population of various origins were obtained from the Exome Aggregation Consortium database (ExAC) (http://exac.broadinstitute.org/) and from the catalogue of human genomic variation, ISB release Kaviar (https://www.systemsbiology.org/research/isb-releases-kaviar-worlds-largest-public-catalog-of-human-genomic-variation/). Variants were numbered in the text and figures according to the sequence NM_000719.6 as most of the previous published variants.

### Cav1.2 subunit expression vectors and mutagenesis

Truncated and WT rabbit Ca_v_1.2 cardiac isoform Ca_v_α_1c_ (NM_001136522), Ca_v_β_2_ (NM_001082396.1) and Ca_v_α_2_δ_1_ (NM_001082276) complementary DNAs (cDNAs) subcloned into pCARDHE, pBH17 and pCA1S, respectively, were gifts from Dr G.S. Pitt (Department of Medicine, Division of Cardiology, Duke University Medical Center, Durham, NC, USA). Mutants were generated using the QuikChange II XL Site-Directed Mutagenesis Kit (Stratagene, USA) according to the manufacturer’s instructions. Primers designed for mutagenesis are available upon request. All plasmids were checked by sequencing.

### Transfections

T25 cm^2^ flasks of TsA-201 cells at 80% confluency were transiently co-transfected using X-tremeGene 9^®^ mix reagent (Roche Diagnostics, IN, USA) with 0.7 µg of each subunit of voltage-gated calcium channel (Ca_v_α_1c_, Ca_v_β_2b_ and Ca_v_α_2_δ_1_ subunits, ratio1:1:1). All transfections included 0.2 µg of cDNA encoding CD8 antigen and 0.1 µg of cDNA encoding GFP as a reporter gene. For the co-expression assays using cleaved CT constructs, the fragment coding for the C-terminal part has been introduce in pIRES vector co-expressing CD8. For patch clamp experiments, anti-CD8 beads (Dynal^®^, Oslo, Norway) were used. Only cells decorated with anti-CD8 beads that were concomitantly green (GFP) were analysed.

### Electrophysiology

Whole-cell currents were measured at room temperature (22–23 °C) using a VE-2 amplifier (Alembic Instrument, USA). The internal pipette solution was composed of (in mmol/L) 60 CsCl, 70 Cs-aspartate, 1 MgCl_2_, 10 HEPES, 11 EGTA and 5 Mg-ATP, pH 7.2, with CsOH. The external solution contained (in mmol/L) 130 NaCl, 5.6 CsCl, 5 BaCl_2_ or 20 mM CaCl_2_, 1 MgCl_2_, 10 HEPES and 11 D-glucose, pH 7.4, with NaOH. Data were analysed using pClamp software, version 10.2 (Axon Instruments, Union City, California, USA). Calcium and barium current densities (pA/pF) were calculated by dividing the peak current by the cell capacitance. Activation curves and steady-state inactivation curves were fitted with the following single Boltzmann equation: y = 1/(1 + exp [(*V*h − *V*_50_)/k]), in which *y* is the normalised conductance or peak current at a given holding potential (*V*h); *V*_50_ is the voltage at which half of the channels are activated (*V*_50_, act) or inactivated (*V*_50_, inact) respectively, and *k* is the slope factor. Calcium-dependent inactivation (CDI) and voltage-dependent inactivation (VDI) in presence of extracellular calcium or barium respectively were calculated at the percentage of current decreased at the end of the 200 ms test pulse.

### Statistical analysis

Data are presented as means ± S.E.M. Unpaired, two-tailed Student’s *t*-test was used to compare the means; *p* < 0.05 was considered significant.

## Electronic supplementary material

Supplementary information
