# Supplementary material for: An African loss-of-function CACNA1C variant p.T1787M associated with a risk of ventricular fibrillation
Source: Sci Rep. 2018 Oct 2;8:14619. doi: 10.1038/s41598-018-32867-4 (PMC6168548; doi:10.1038/s41598-018-32867-4)
Supplement: Supplementary file 1 — Supplementary information [file 41598_2018_32867_MOESM1_ESM.docx]

**An African loss-of-function *CACNA1C* variant, p.T1787M, associated with a risk of ventricular fibrillation**

Short title: A loss-of-function *CACNA1C* variant associated with ventricular fibrillation

Malorie Blancard^1,2^, Amal Debbiche^1,2^, Koichi Kato^1,2^, Christelle Cardin^3^, Guichard Sabrina^4^, Estelle Gandjbakhch^1,2,5^, Vincent Probst^6^, Michel Haissaguerre^7,8^, Fabrice Extramiana^9^, Mélèze Hocini^7,8^, Geoffroy Olivier^10^, Antoine Leenhardt^9^, Pascale Guicheney*^1,2^, Jean-Sébastien Rougier*^4^.

^1^ INSERM, UMR_S1166, Paris, France.

^2^ Sorbonne University, Institute of Cardiometabolism and Nutrition (ICAN), Paris, France.

^3^ University Hospital Rangueil, Toulouse, France.

^4^ University of Bern, Institute of Biochemistry and Molecular Medicine, Bern, Switzerland.

^5^ AP-HP, Hôpital Pitié-Salpêtrière, Département de Cardiologie, Paris, France.

^6^ CHU Nantes, L'institut du thorax, Service de Cardiologie, Nantes, France.

^7^ L'Institut de Rythmologie et Modélisation Cardiaque (LIRYC), Université de Bordeaux, Bordeaux, France.

^8^ Inserm U1045 CRCTB, Université de Bordeaux, Bordeaux, France.

^9^ AP-HP, Hôpital Bichat, Département de Cardiologie, Centre de Référence des Maladies Cardiaques Héréditaires, Paris, France.

^10^ CHU Sud Réunion, La Réunion, France.

*Drs. Guicheney and Rougier contributed equally as senior authors.

Corresponding author: Dr Jean-Sebastien Rougier

Address: Institute of Biochemistry and Molecular Medicine, Bühlstrasse 28, 3012 Bern, Switzerland

e-mail: jean-sebastien.rougier@ibmm.unibe.ch

Tel: +41 31 631 53 14

SUPPLEMENTAL

**Materials and Methods**

**Electrophysiological study of the variant of Na_v_1.5**

*SCN5A* p.Leu618Phe variant was created by site-directed mutagenesis using Quikchange II XL kit. HEK293 cells were transiently transfected with 0.5 µg of pcDNA3.1-hH1a WT or L618F plasmid in Q1077del background by jetPEI transfection reagent according to the manufacturer’s instructions. Sodium current recordings were performed 36-48 hours after transfection by using the whole-cell patch clamp technique. Through this procedure, pipettes were filled with the intra-pipette solution containing (in mmol/L) NaCl 5, MgCl_2_ 2, CaCl_2_ 1, EGTA 15, HEPES 1, MgATP 4, and CsCl 130 (pH was adjusted to 7.2 with CsOH). Pipette resistances were around 1.0-1.5 MΩ. The bath solution contained (in mmol/L): NaCl 135, KCl 4, CaCl_2_ 1, MgCl_2_ 2.5, HEPES 1, and glucose 1 (pH was adjusted to 7.4 with CsOH).

**Biochemistry experiments**

**Transfections**

T25 cm^2^ flasks of TsA-201 cells at 80% confluency were transiently co-transfected using X-tremeGene 9^®^ mix reagent (Roche Diagnostics, IN, USA) with 0.7 µg of each subunit of voltage-gated calcium channel (Ca_v_α_1c_, Ca_v_β_2b_ and Ca_v_α_2_δ_1_ subunits, ratio1:1:1). Cells were used 48 hours after transfection.

**Surface biotinylation assay**

Cells were treated for 30 minutes at 4°C with 4 ml biotin per 10-cm dish (1 mg/ml; EZ link Sulfo-NHS-SS-Biotin; Pierce, Rockford, USA), washed three times with cold PBS containing 200 mM glycine and lysed with 1-ml/dish of lysis buffer. Fifty µl of streptavidin sepharose beads (GE Healthcare Europe, Glattbrugg, Switzerland) were added to 1 mg of TsA-201 cell lysate and incubated for 2 hours on a wheel at 4°C. The beads were washed five times with lysis buffer, and resuspended in sample buffer (Invitrogen, Basel, Switzerland). Eluted proteins were analyzed by Western blot.

**Western blots**

T25 cm^2^ TsA-201 cell flasks were lysed in 1 mL of lysis buffer (50 mmol/L HEPES pH 7.4, 150 mmol/L NaCl, 10% glycerol, 1% triton, 1 mmol/L EGTA supplemented with protease inhibitors). Protein concentration was systematically determined by performing a Braford assay (Coo protein dosage kit; Interchim, Montluçon, France). Seventy micrograms of proteins were loaded on SDS-PAGE 5-15% gradient gel. Protein transfer was done with the dry system transfer i-blot® from Invitrogen (Invitrogen, Basel, Switzerland). Immunoblotting was done using the snap-id® system of Millipore (Millipore, Zug, Switzerland).

**Antibodies**

Antibodies against Ca_v_α_1c_ (ACC003) (Alomone, Jerusalem, Israel), Ca_v_β_2_ (ab54920) and Ca_v_α_2_δ_1_ (ab2864) (Abcam, Cambridge, United Kingdom) were used at a dilution of 1/200 for Ca_v_1.2α1 and Ca_v_β_2b_ and 1/1000 for Ca_v_α_2_δ_1_. Polyclonal antibody raised against actin (A-2066) was purchased from Sigma-Aldrich (Sigma-Aldrich Chemie, Postfach, Switzerland) and used at a dilution of 1/1000. Monoclonal antibody raised against Na/K ATPase was purchased from Abcam (ab7671) (Abcam, Cambridge, United Kingdom) and used at a dilution of 1/1000. Fluorescent secondary antibodies at 1/10,000 dilution were used and detection was performed using the LICOR system^®^ (Lincoln, USA).

**Supplementary Figure S1**

**
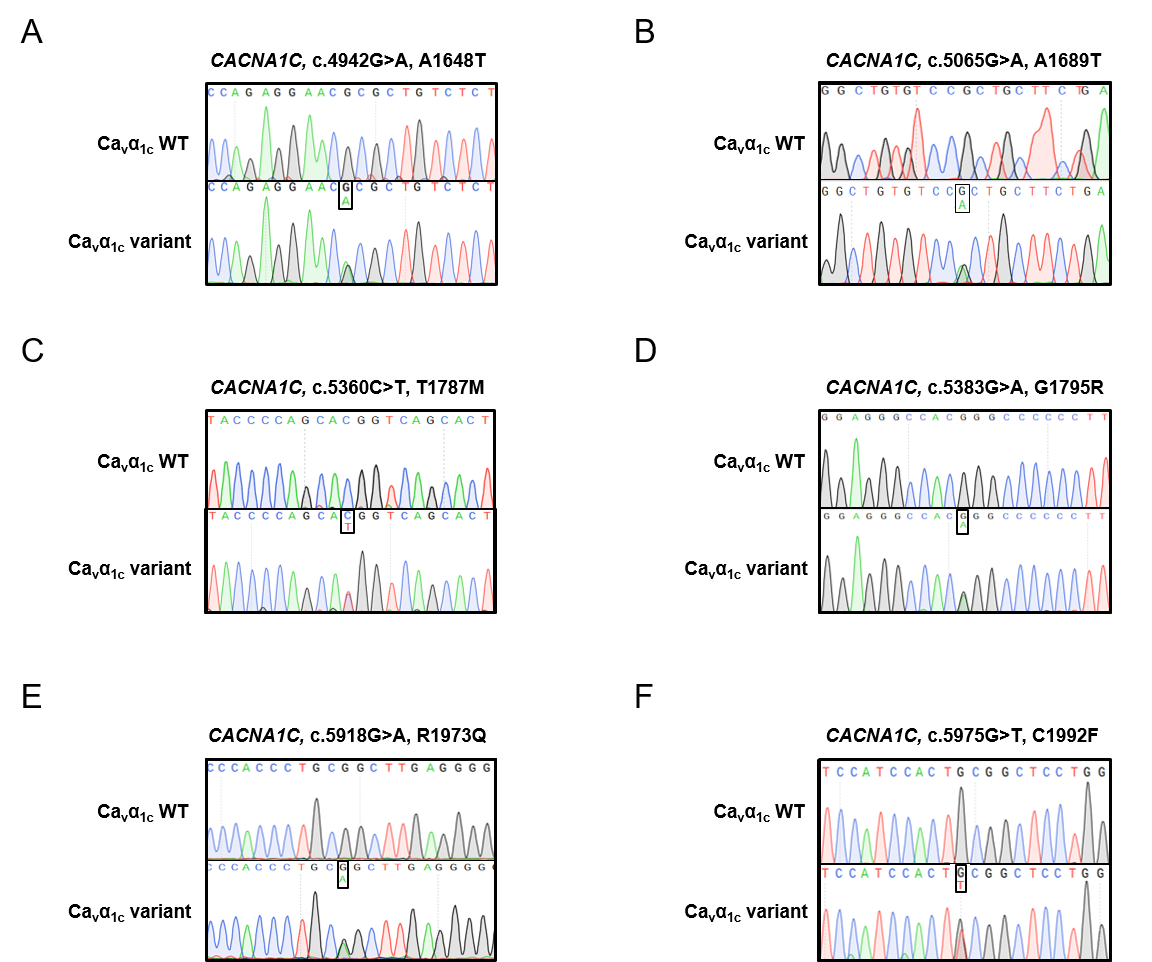
**

**Chromatograms of each variant investigated in this study.**

**Supplementary Figure S2**

**
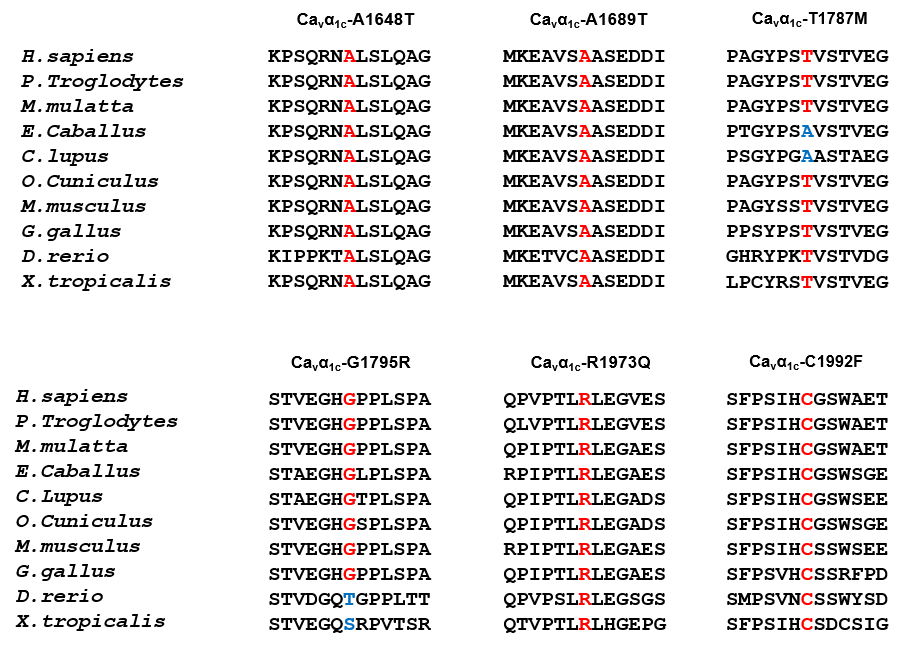
**

**Evolutionary conservation of the mutated amino acids**

The alignment of the calcium channel subunit Ca_v_α_1c_ sequences surrounding the missense variants was performed using the following Uniprot accession numbers: *Homo sapiens*: Q13936, *Pan troglodytes*: H2Q573, *Macaca mulatta*: F7HT59, *Equus caballus*: F6YFJ5, *Canis lupus familiaris*: F1Q382, *Oryctolagus cuniculus*: P15381, *Mus musculus*: Q01815, *Gallus gallus*: F1NJP7, *Danio rerio*: Q5TZF1, *Xenopus tropicalis*: F6S8W3.

**Supplementary Figure S3**

**
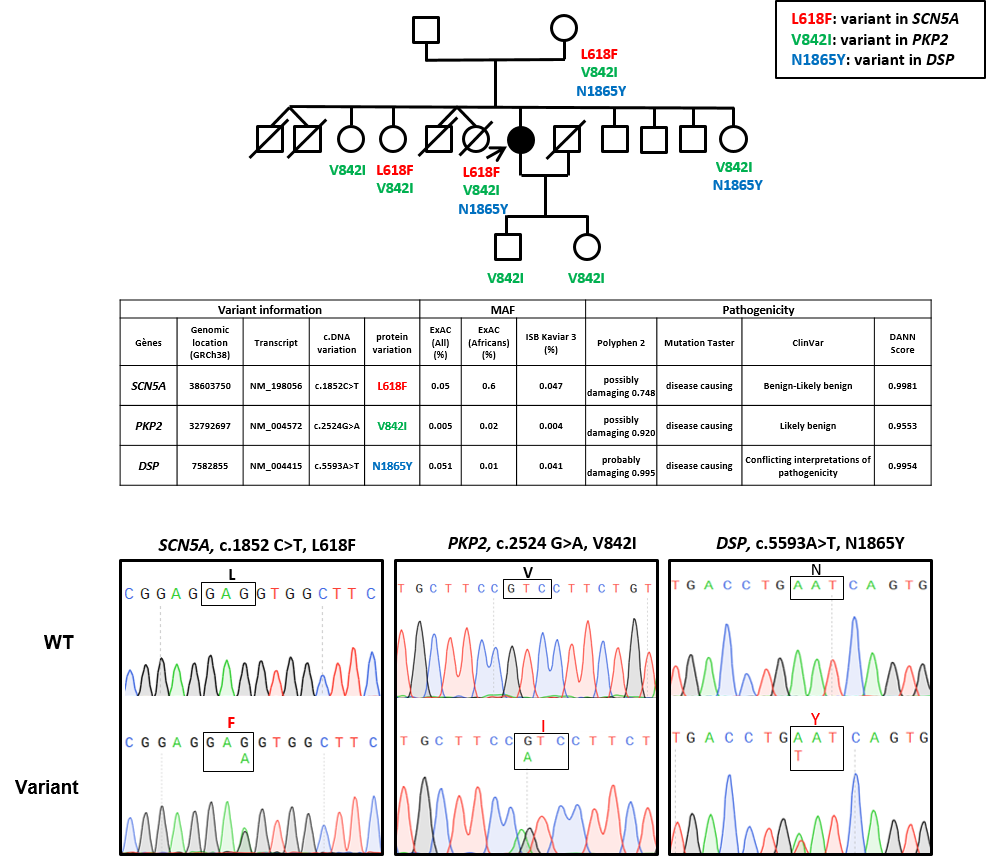
**

**Additional missense variants identified in Case 2 and family members**

MRI performed in two sisters who regularly practice sport (II-3, II-4, 54 and 53 years old) and another sister (II-12, 41 years old), and in her son (III-1) did not show any sign of ARVC. The mother (I-2) is 72 years old; she presented with hypertension and diabetes and showed a mild dilatation of the right ventricle at echocardiography. Family member QTc intervals were in a normal range at rest [390-410 ms] and during exercise.

**Supplementary Figure S4**


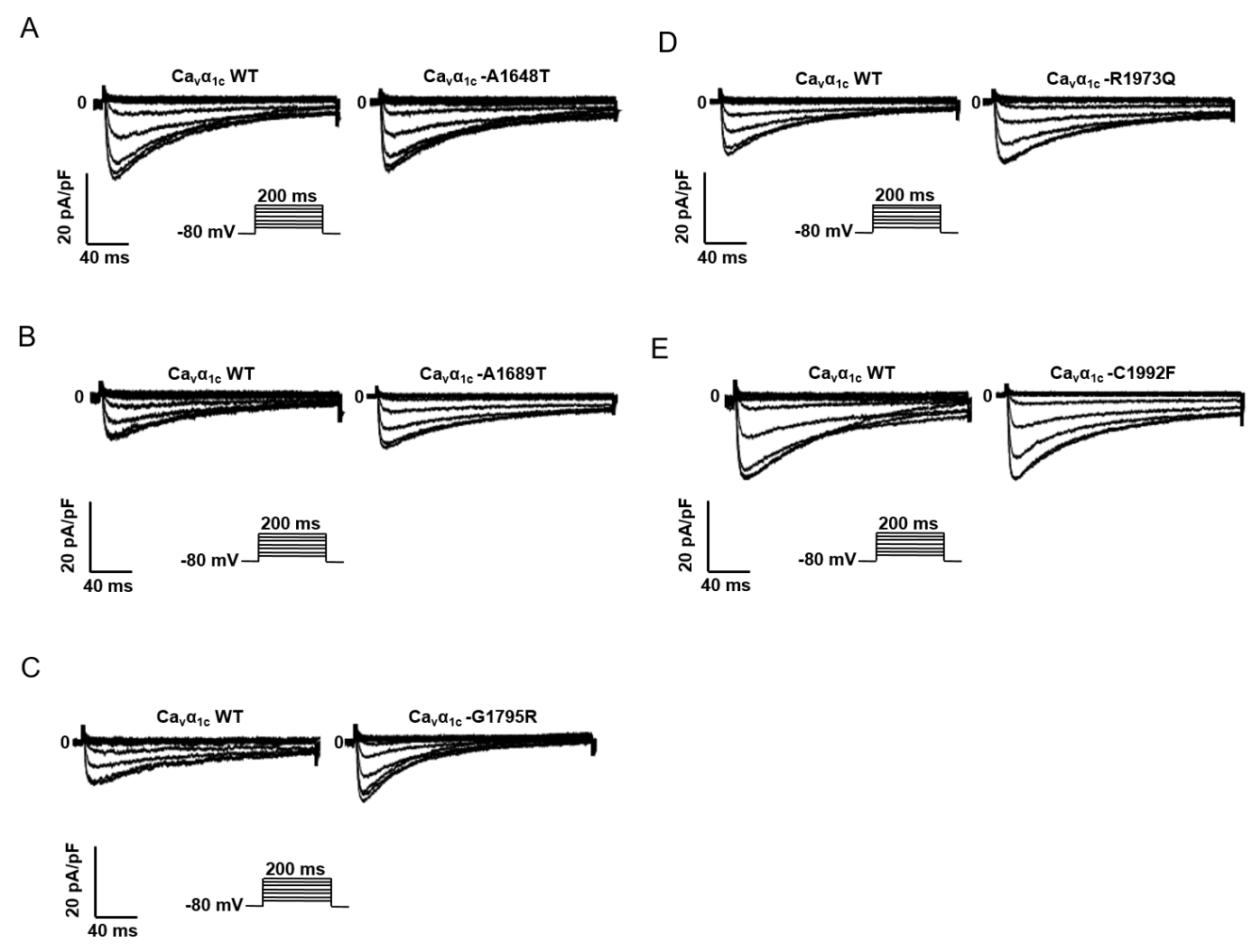


**Ca_v_α_1c_ variants: calcium currents**

A to E: representative whole-cell calcium current traces showing the absence of an effect of different Ca_v_α_1c_ variants.

**Supplementary Figure S5**

**
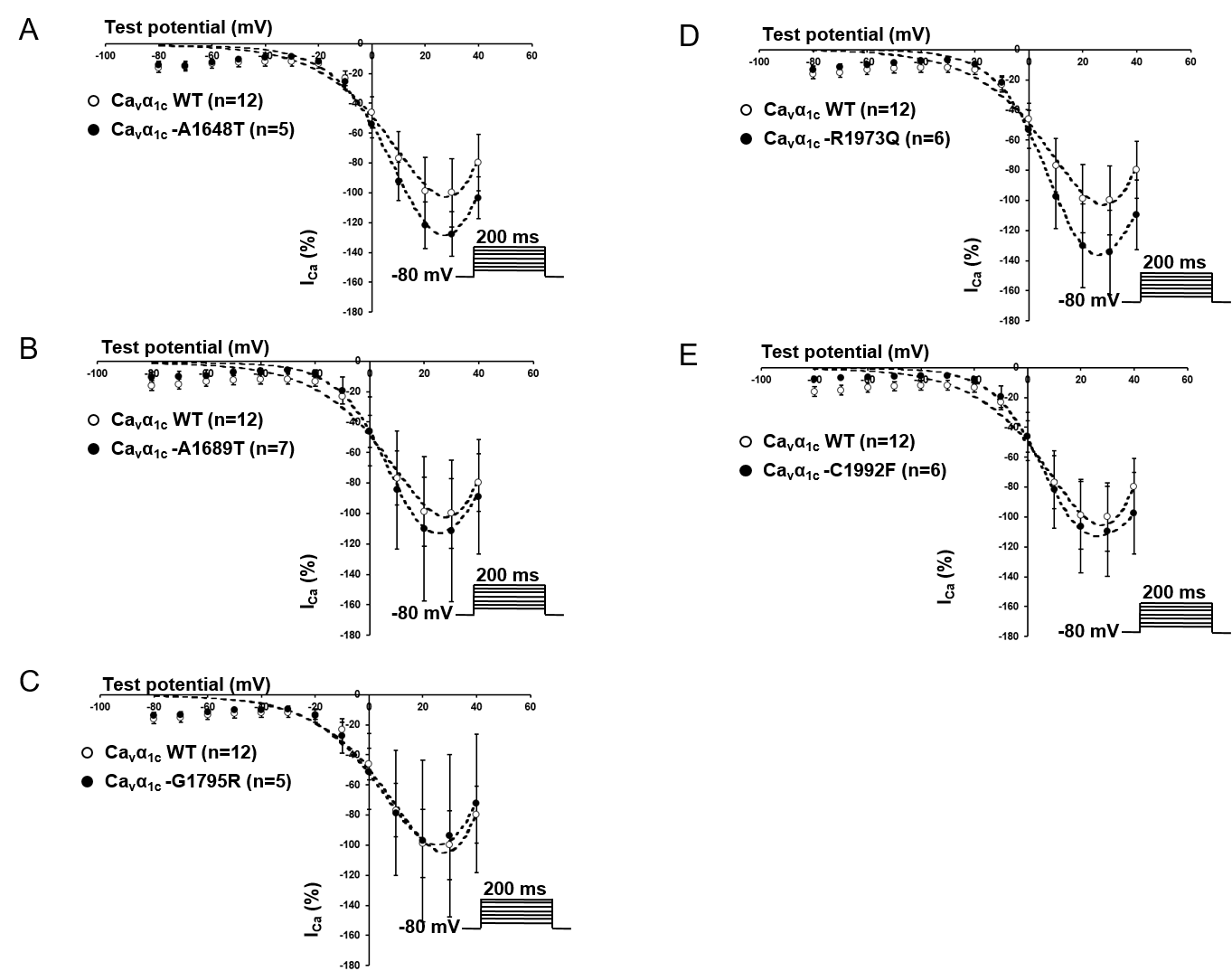
**

**Current-voltage relationships of calcium channel variants in the presence of extracellular calcium**

A to E: current-voltage relationships in cells transfected with either WT (○) or Ca_v_α_1c_ variants (●). The number of cells is indicated in parentheses.

**Supplementary Figure S6**


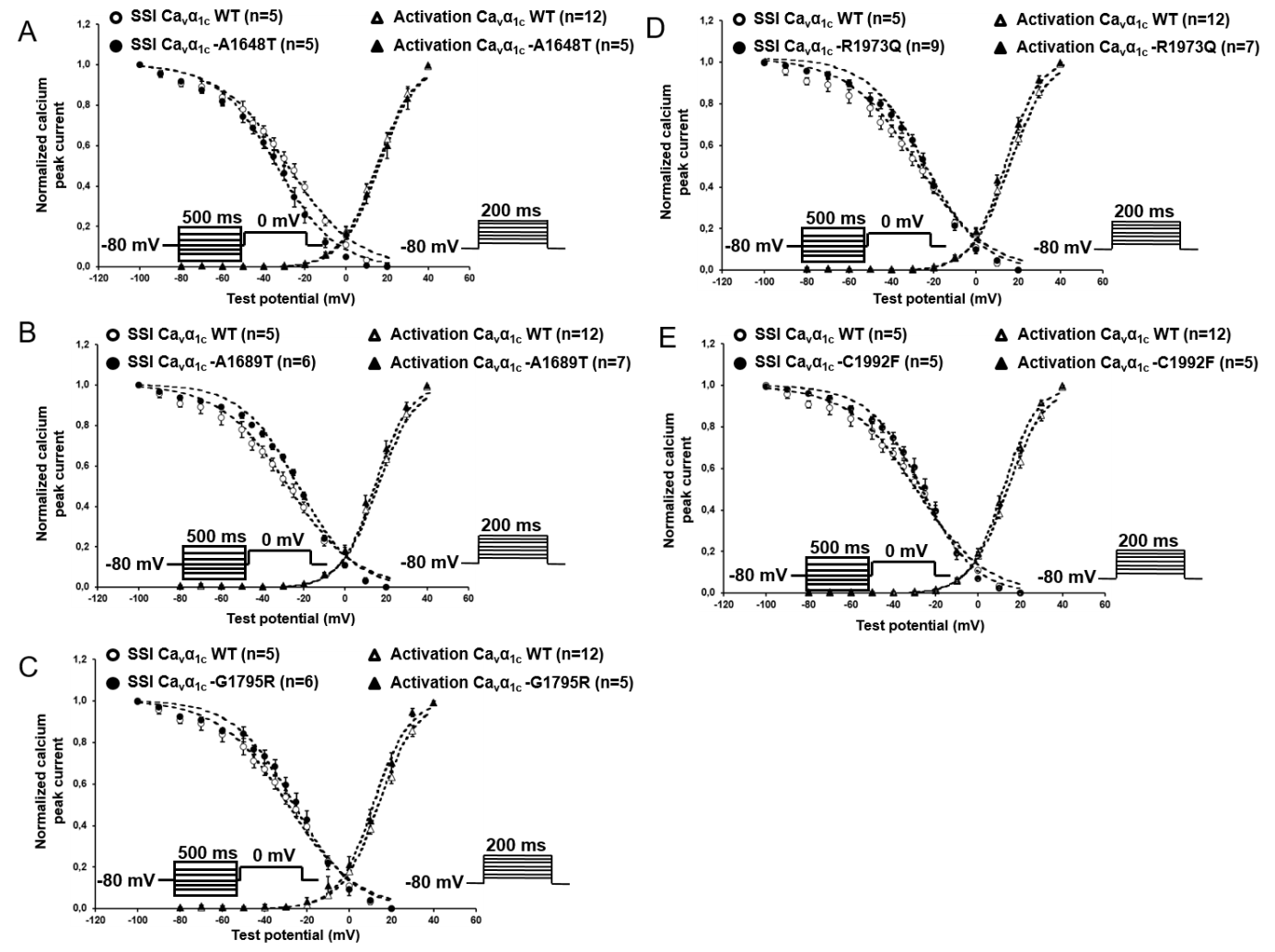


**Biophysical properties of voltage-gated calcium channel variants in the presence of extracellular calcium**

A to E: steady-state inactivation and activation curves of either WT (white symbol) or Ca_v_α_1c_ variants (back symbol) showing no major alterations. The number of cells is indicated in parentheses.

**Supplementary Figure S7**

**
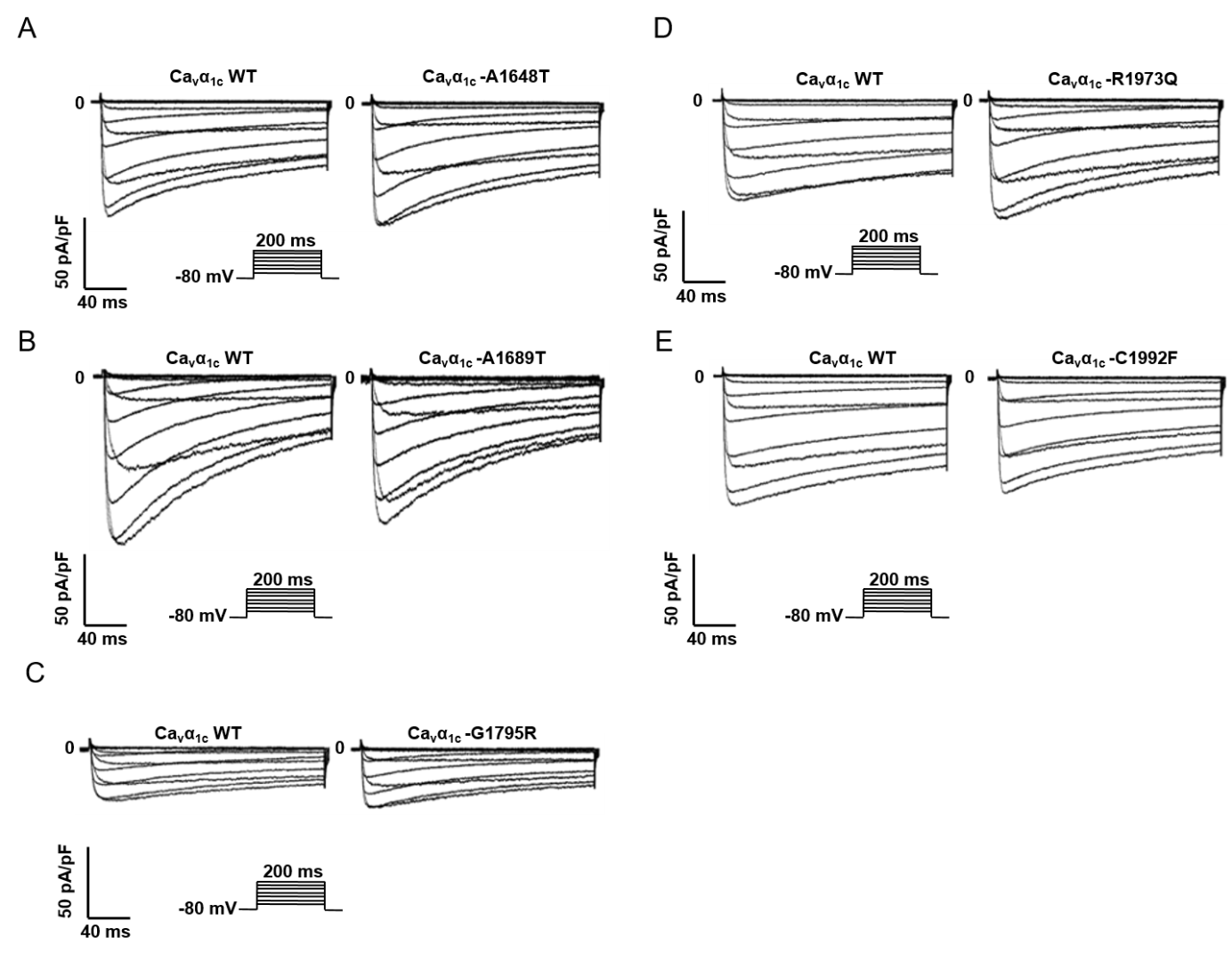
**

**Ca_v_α_1c_ variants: barium currents**

A to E: representative whole-cell calcium current traces showing the absence of an effect of different Ca_v_α_1c_ variants.

**Supplementary Figure S8**


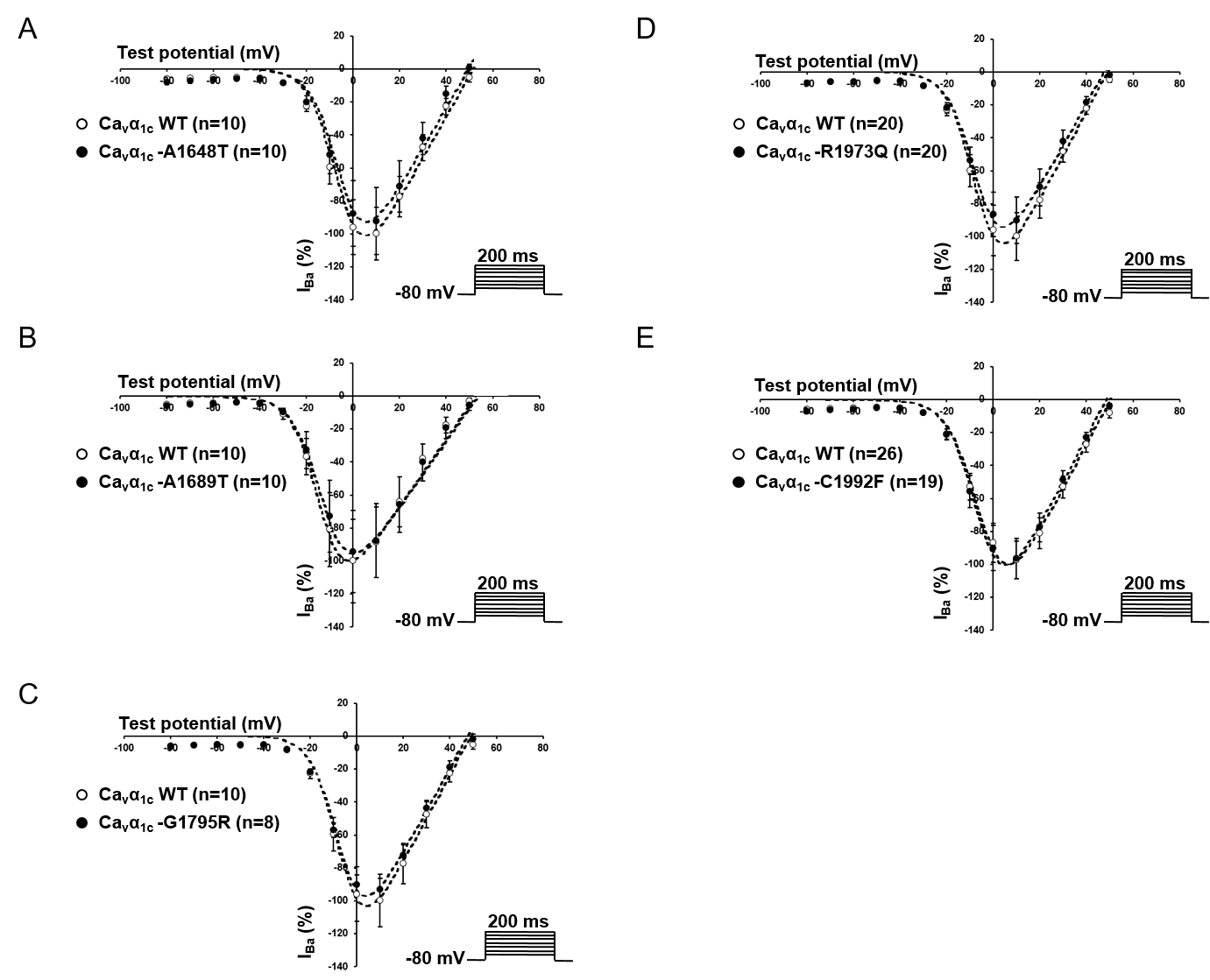


**Current-voltage relationships of calcium channel variants in the presence of extracellular barium**

A to E: current-voltage relationships in cells transfected with either WT (○) or Ca_v_α_1c_ variants (●). The number of cells is indicated in parentheses.

**Supplementary Figure S9**


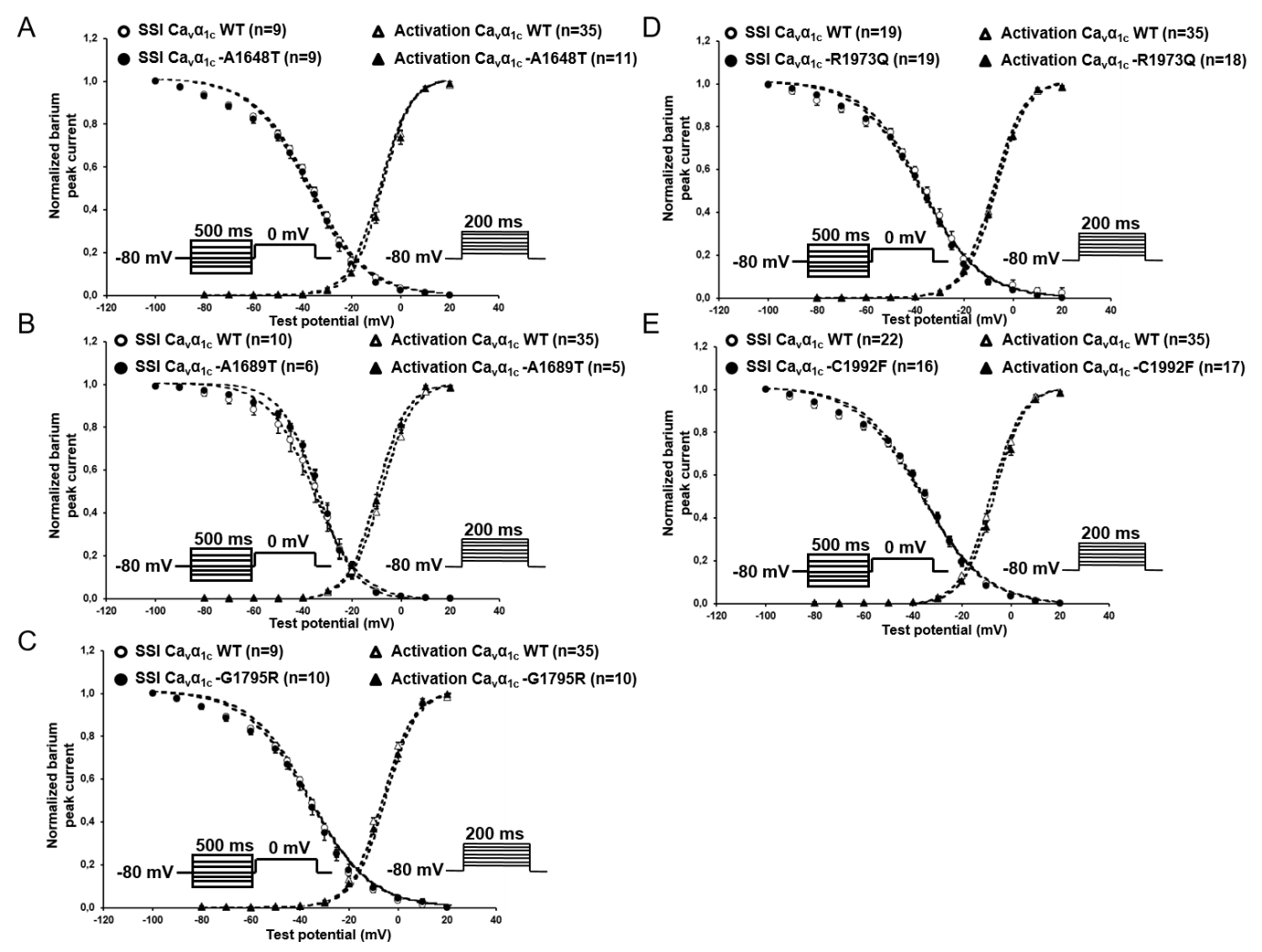


**Biophysical properties of voltage-gated calcium channel variants in the presence of extracellular barium**

A to E: steady-state inactivation and activation curves of either WT (white symbol) or Ca_v_α_1c_ variants (back symbol) showing no major alterations. The number of cells is indicated in parentheses.

**Supplementary Figure S10**


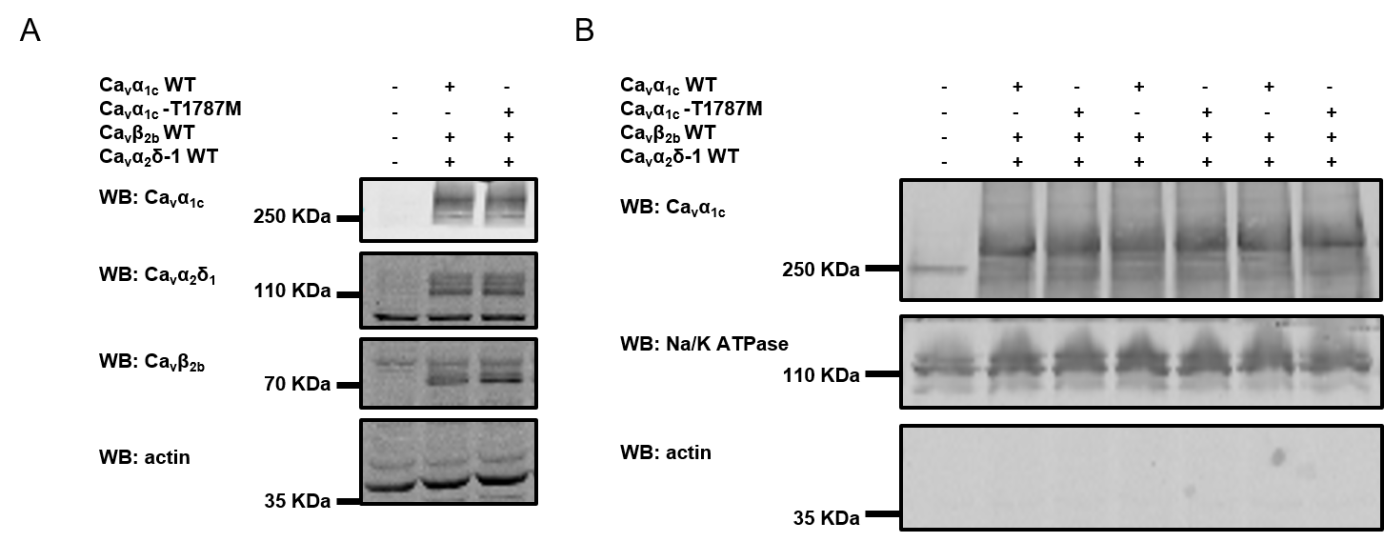


**Whole-cell and surface expression of calcium channel subunits in the presence of the Ca_v_α_1c_-T1787M variant**

A: Representative western blot of whole-cell lysates showing no modification of expression of the three Ca_v_1.2 subunits when the Ca_v_α_1c_-T1787M variant is expressed. B, western-blot of biotinylated fractions of Ca_v_α_1c_ subunits showing no modification of expression at the cell surface when the Ca_v_α_1c_-T1787M variant is expressed.

**Supplementary Figure S11**


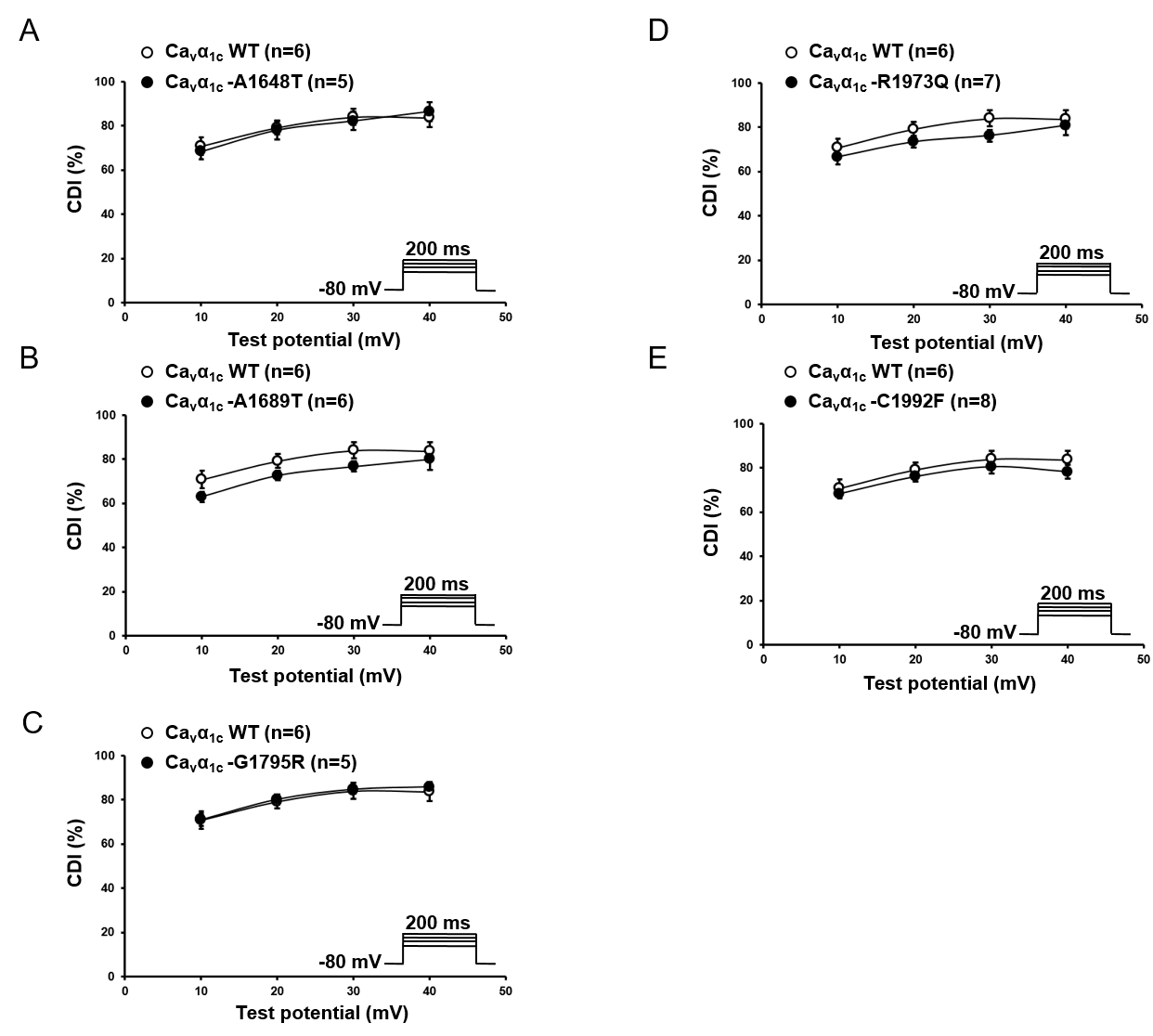


**Calcium-dependent inactivation of voltage-gated calcium channel variants**

A to E: calcium-dependent inactivation (CDI) with either WT (○) or Ca_v_α_1c_ variants (●) showing no alteration. The number of cells is indicated in parentheses.

**Supplementary Figure S12**


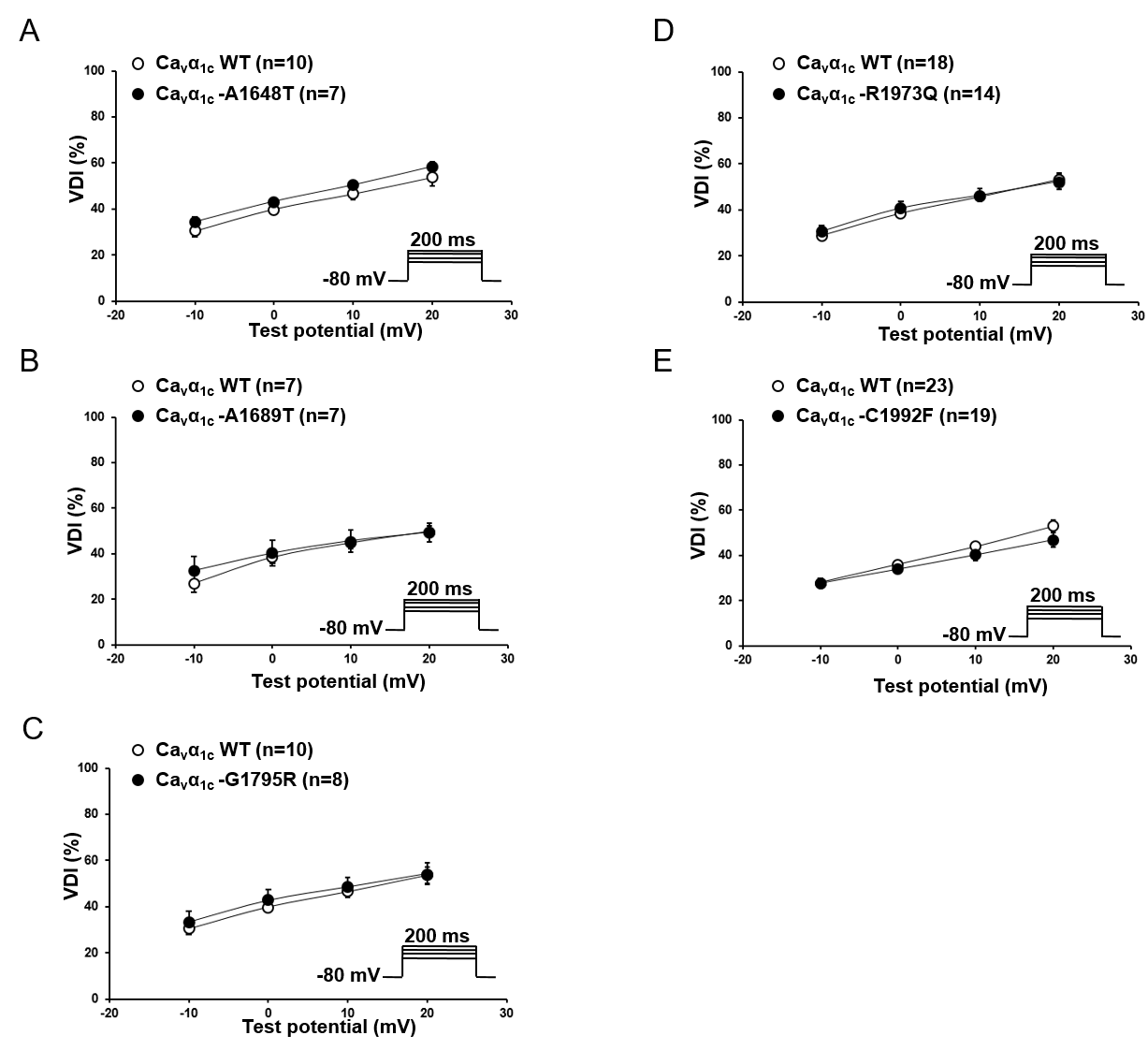


**Voltage-dependent inactivation of voltage-gated calcium channel variants**

A to E: voltage-dependent inactivation (VDI) with either WT (○) or Ca_v_α_1c_ variants (●) showing no alteration. The number of cells is indicated in parentheses.

**Supplementary Figure S13**


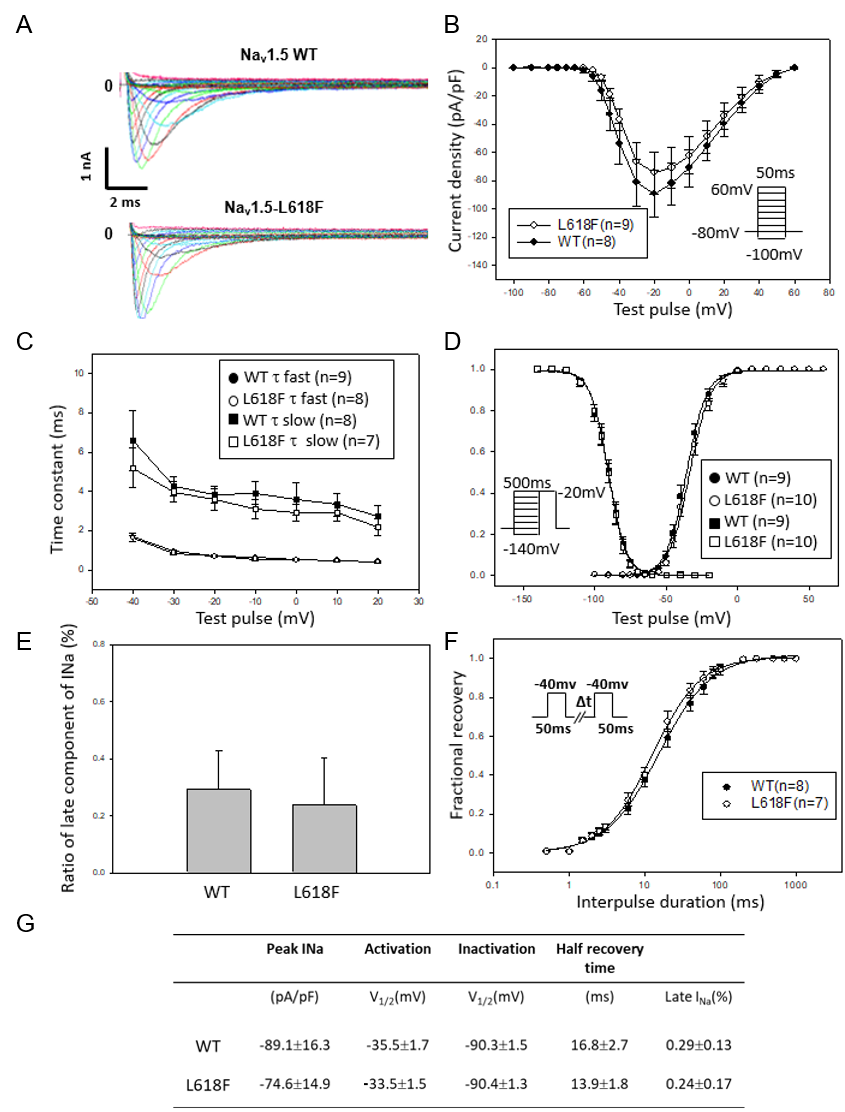


**Electrophysiological study of the Na_v_1.5-L618F variant**

A: Representative original current traces of Na_v_1.5 WT and Na_v_1.5 L618F channels. B: current-voltage relationships reveal no statistical difference in peak current densities. C: Inactivation time constants did not show statistical difference between Na_v_1.5 WT and Na_v_1.5 L618F. D: Voltage-dependent activation and inactivation curves. In both activation and inactivation, Na_v_1.5 L618F channels did not shift the voltage dependence compared to the WT channels. E: Late tetrodotoxin-sensitive (30 µM) sodium currents, measured at the end of 500 ms long duration test pulse, did not shown any alteration between the wild-type and variant channel. F: Recovery from inactivation from WT and Na_v_1.5 L618F variant channels. G: table summarising all of the parameters measured showing no alteration between the WT and the Na_v_1.5 L618F variant channel.
